# Poly(2-(diethylamino)ethyl methacrylate)-Functionalized Carbon Nanodots as Theranostic Platforms for siRNA Delivery and Survivin Silencing in Triple-Negative Breast Cancer

**DOI:** 10.1021/acs.biomac.5c00267

**Published:** 2025-05-10

**Authors:** Paola Varvarà, Gennara Cavallaro, Nicolò Mauro

**Affiliations:** † Laboratory of Biocompatible Polymers, Department of “Scienze e Tecnologie Biologiche Chimiche e Farmaceutiche” STEBICEF, 18998 University of Palermo, Via Archirafi 32, 90123 Palermo, Italy; ‡ Fondazione Veronesi, Piazza Velasca 5, 20122 Milano, Italy

## Abstract

This study describes the development of carbon nanodot (CDs)-based theranostic nanocarriers that integrate gene silencing with fluorescence imaging. Nitrogen- and sulfur-doped CDs were functionalized through controlled radical surface polymerization of 2-(diethylamino)­ethyl methacrylate (DEAEMA), yielding self-tracking, cationic siRNA carriers CDs-pDEAEMA. The functionalization of CDs enhanced their fluorescence, broadening the emission spectrum toward the biologically transparent window. Fluorescent CDs-pDEAEMA effectively bound siRNA, remaining stable under physiological conditions, while in vitro studies proved their hemocompatibility and cytocompatibility on human dermal fibroblasts. Moreover, the ability to deliver BIRC5 siRNA was demonstrated in MDA-MB-231, successfully transfecting triple-negative breast cancer cells and resulting in an 80% reduction in the anti-apoptotic protein survivin. Furthermore, uptake studies demonstrated that the theranostic CDs are efficiently internalized in tumor cells and are clearly detectable by fluorescence imaging in the red region. These findings highlight the potential of CDs-pDEAEMA as an advanced theranostic tool for real-time tracking of siRNA therapy of breast cancer.

## Graphical abstract

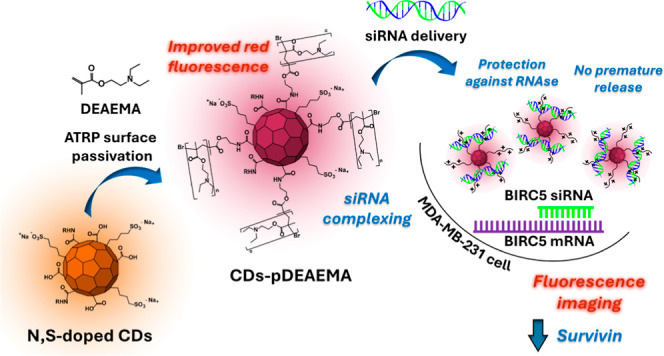

## Introduction

Despite advances in early detection and therapeutic strategies, breast cancer (BC) remains a major global health challenge, representing the most prevalent malignancy diagnosed among women.^1–3^ One of the greatest hurdles in BC management lies in its complexity, since it displays an array of subtypes with distinct genetic and molecular characteristics.^4–6^ This inherent heterogeneity, coupled with the inter- and intra-individual variability, complicates treatment, as different cancers respond differently to available therapies.^7^ As a result, conventional approaches are not always effective, particularly against more aggressive and treatment-resistant forms such as triple-negative breast cancer (TNBC).^8–10^ Personalized medicine, which adapts treatment to the patient’s profile, tumor characteristics, and response to previous therapies, has emerged as tailored strategy to overcome these obstacles.^11,12^ In this context, small interfering RNA (siRNA) therapy represents a versatile approach to cancer treatment, offering a method for silencing specific genes involved in tumor progression. These interfering fragments can be used to target proteins responsible for promoting cancer cell survival, proliferation, and metastasis, potentially modulating multiple stages of the disease.^13–15^ For instance, a key target for siRNA therapy is the inhibition of anti-apoptotic proteins such as Bcl-2 or survivin, which prevent cancer cells from undergoing programmed cell death.^16–19^ Additionally, siRNA therapy offers high specificity, reducing the likelihood of off-target effects compared to traditional chemotherapeutic agents, which often cause damage to healthy tissues and lead to severe side effects. However, the clinical application of siRNA requires the refinement of efficient strategies to accomplish stability in the bloodstream and the delivery to tumor cells.^13,20^ Thus, the effort to tackle the combined challenges of siRNA delivery and BC heterogeneity has driven the exploration of innovative nanomaterials as multimodal delivery platforms.^21,22^

Carbon dots (CDs) are a class of carbon-based nanomaterials that have gained significant attention due to their unique optical properties, including strong luminescence, photostability, and efficient near-infrared (NIR) photothermal conversion that make them ideal for sensing, imaging, and therapeutic applications, such as fluorescence-based detection and photothermal treatment of cancer cells and tissues.^23,24^ Moreover, their small size and high surface area also allow for the efficient loading of chemotherapeutics with diverse chemical natures. These fascinating properties explain why these nanomaterials have emerged as compelling options for a range of biomedical applications, especially in theranostics, which integrates therapy and real-time diagnostic imaging.^25^ One of the major advantages of CDs is their versatility, due to the chance to modify their properties through surface functionalization.^26,27^ Indeed, as the surface of CDs provides a highly reactive platform for the covalent attachment of small molecules or macromolecules, it is possible to design functional coatings that impact their fluorescence intensity, emission wavelength, and delivery capabilities, tuning their properties to meet specific therapeutic needs.

Considering this scenario, this study focused on designing an advanced multifunctional platform that combines gene silencing of the anti-apoptotic protein survivin with real-time imaging capabilities. Specifically, N,S-doped CDs with uniform size and multicolor emission with proven imaging features and therapeutic potential against breast cancer cells^28^ were engineered through ATRP surface polymerization of 2-(diethylamino)­ethyl methacrylate (DEAEMA) monomers to pursue a self-tracking complexing agent for siRNA therapy (CDs-pDEAEMA). After chemical–physical characterization, the effects of CDs passivation on the optical properties were evaluated and the device’s ability to effectively interact with nucleic acids, forming siRNA delivery complexes that are stable and safe in the bloodstream, was assessed.

Thus, the potential of this hybrid theranostic tool for delivering BIRC5 siRNA in the triple negative BC cell line MDA-MB-231 was explored by investigating cell viability, gene-silencing efficiency, and cellular uptake.

## Materials and Methods

### Materials

Urea (99%), citric acid (100%), indocyanine green (ICG, 97%), *N*,*N*′-dicyclohexylcarbodiimide (DCC, 99.5%), *N*-hydroxysuccinimide (NHS, 99.5%), ethanolamine (99%), triethylamine (TEA, 99.5%), α-bromoisobutyryl bromide (BIBB, 99%), 2-(diethylamino)­ethyl methacrylate (DEAEMA, 99.5%), copper­(I) bromide (CuBr, 99.5%), 2,2 -bipyridyl (Bpy, 99.5%), anhydrous *N*,*N*-dimethylformamide (DMF, >99.5%), diethyl ether (>99.5%), methanol (>99.5%), deuterium oxide (D_2_O), acetonitrile (ACN, (99.5%)), Trizma base (99%), HEPES (99%), sodium dodecyl sulfate (98.5%), sodium chloride (99.5%), ethylenediaminetetraacetic acid tetrasodium salt dihydrate (99%), PEO standards for SEC (ReadyCal-Kit PEO/PEG), Triton X-100 (99%), glucose (99.5%), RNAse A from bovine pancreas, heparin sodium salt from porcine intestinal mucosa (>180 USP units mg^–1^) were purchased from Merck. Water for molecular biology (sterile, nuclease-free, phosphatase, and protease free) was purchased from the former supplier and used for the preparation and handling of complexes. Cellulose acetate 0.45 μm, nylon 0.45 μm Acrodisc, and Glass Fiber Pall 10 μm syringe filters were purchased from VWR. Sodium dihydrogen phosphate (NaH_2_PO_4_), acetic acid, and Sephadex SEC resins and dialysis tubing were purchased from Carlo Erba (Italy).

BIRC5 siRNA (Silencer pre-designed siRNA) targeting the gene responsible for survivin protein (BIRC5), non-targeting scrambled siRNA (Silencer Negative Control #1 siRNA), Lipofectamine Transfection reagent and RediPlate 96 RiboGreen RNA Kit were purchased from Thermo Fisher. Cy5 siRNA, used as labeled non-targeting scrambled siRNA (MISSION siRNA Fluorescent Universal Negative Control #1, Cyanine 5) and BCA Protein Assay Kit were purchased from Merck. BIRC5 siRNA sequence (5′- > 3′): sense CCACUUCCAGGGUUUAUUCtt; antisense GAAUAAACCCUGGAAGUGGtg. The sequences of Silencer Negative Control #1 siRNA and of MISSION siRNA Fluorescent Universal Negative Control #1, Cyanine 5 are proprietary. The CellTiter 96 AQueous One Solution Cell Proliferation Assay (MTS) was obtained from Promega while the Human Survivin ELISA kit was purchased from antibodies.com and used following the manufacturer’s instructions.

### Carbon Nanodots Synthesis (CDs)

The CDs were synthesized as previously described, using urea (11.56 g, 57.7 mmol), citric acid (36.95 g, 57.7 mmol), and ICG (0.1 g, 0.129 mmol) as precursors and dissolving them in DMF (100 mL).^28^ The reaction mixture was sealed in an still autoclave (Buchiglas AG) and heated under solvothermal conditions at 170 °C for 6 h (rump-time of 30 min). Afterward, the solvent was evaporated under reduced pressure (25 mbar, 80 °C), leaving a brown solid residue. The retrieved solid was then redispersed in 150 mL of ultrapure water by sonication (15 min, 5 cycles), and the resulting aqueous dispersion of CDs was filtered first through paper and then using a 0.45 μm membrane filter. The dispersion was further purified by using size exclusion chromatography (SEC), employing a column packed with Sephadex G15 and G25 in series. The collected fractions were analyzed and combined according to their UV/FL absorption spectra, resulting in a sample of 5.2 ± 0.3 nm in diameter with multicolor emission. The obtained CDs were analyzed using Fourier Transform Infrared (FT-IR) spectroscopy (PerkinElmer Spectrum Two), collecting data in the range of 4000–400 cm^–1^.

FT-IR: 3420 cm^–1^, ν (O–H) alcohol; 3200 cm^–1^, ν (N–H) amine; 1715 cm^–1^, ν_as_ (C=O) carboxylic acid; 1640 cm^–1^, ν (C=O) amide I; 1410 cm^–1^, ν_as_ (O–SO_3_−) sulfonate/sulfoxide; 871 cm^–1^, ν (C–S) sulfonate/sulfoxide.

### ATRP Polymerization on Carbon Dots: Synthesis of CDs-pDEAEMA

The synthesis of CDs-pDEAEMA involved a 3-step pathway without isolation of intermediates. First, 40 mg of CDs was dissolved in 8 mL of previously degassed aDMF, aiding the process with a sonicating bath for 15 min. Then, NHS (36.14 mg, 0.314 mmol) and DCC (64.76 mg, 0.314 mmol) were both dissolved separately in 2 mL of aDMF, added as solutions into the CDs dispersion, and left to react at 40 °C for 4 h. On completion, ethanolamine was added (37.68 μL, 0.622 mmol), the temperature decreased until 25 °C, and the reaction proceeded under stirring for 18 h at room temperature. Before starting the second step, to remove the solid byproduct DCU, the content of the flask was withdrawn with a glass syringe maintaining inert conditions under argon pressure and then filtered through glass fiber and nylon syringe filters in tandem (cutoff 10 and 0.45 μm, respectively) while transferring the filtrate liquid into a Schlenk flask under argon flow. The flask was thus placed into an ice bath, and TEA (87.3 μL, 0.62 mmol) was added. After 10 min under stirring, 77.6 μL (0.62 mmol) of BIBB was slowly dripped into the reaction mixture, and the mixture was maintained in an ice bath for further 15 min before moving the flask to gently reach room temperature and the whole content was kept reacting for 4 h. The last step of the CDs-pDEAEMA synthesis consisted of ATRP polymerization. Under an argon atmosphere, 12 mL of degassed methanol was poured into the flask to reach the aDMF/methanol mixture 1:1 v/v and DEAEMA monomer (1514.4 μL, 7.54 mmol) was added. Finally, 90 mg of CuBr (0.62 mmol) and 392.32 mg (2.52 mmol) were mixed as solids and introduced into the mixture under vigorous stirring and argon bubbling. The reaction proceeded under an inert atmosphere at 50 °C for 24 h, and upon completion, the flask was opened to stop the process. The resulting product was isolated by slowly dripping the reaction mixture into cold diethyl ether, and the brownish-green solid was washed twice with the same solvent. The obtained residue was dissolved in water and purified twice by both dialysis (MWCO = 3.5 kDa) and SEC with a column packed with Sephadex G25, before being freeze-dried.

CDs-pDEAEMA functional groups were explored through Fourier Transform Infrared (FT-IR) spectroscopy. Spectra were acquired using a PerkinElmer Spectrum Two spectrometer over the wavenumber range of 4000–400 cm^–1^. FT-IR: 3425 cm^–1^, ν (O–H) alcohol; 3250 cm^–1^, ν (N–H) amine II, shoulder; 2967 cm^–1^ and 2936 cm^–1^ ν_as_ and ν_s_ (C–H) aliphatic; 1730 cm^–1^, ν (C=O) ester; 1640 cm^–1^, ν (C=O) amide I; 1460 cm^–1^, δ (C–H) aliphatic; 1390 cm^–1^, ν_as_ (O–SO_3_−) sulfonate/sulfoxide; 1377 cm^–1^, ν (C–N), amine; 1150 cm^–1^, ν_as_ (CO–OC) ester; 950 cm^–1^, 1060 cm^–1^ and 1020 cm^–1^, ν_s_ (SO_2_) sulfonate.

^1^H NMR spectroscopy was carried out by employing a Bruker Avance II 400 spectrometer operating at 400.15 MHz. ^1^H NMR CDs-pDEAEMA (D_2_O, 400.15 MHz): δ 1.00–1.16 (3H_DEAEMA_ –CH_2_–C–CH_3_), δ 1.28 (6H_DEAEMA_ N–(CH_2_–CH_3_)_2_); 1.7 (6H_BIB_, (CH_3_)_2_–C–CO−); δ 2.02 (2H_DEAEMA_ −CH_2_–C–CH_3_); δ 3.08 (4H_DEAEMA_ N–(CH_2_–CH_3_)_2_); δ 3.31­(2H_DEAEMA_ –CH_2_–CH_2_–N–(CH_2_–CH_3_)_2_); δ 3.73 (2H_ethanolamine_ −CO–NH–CH_2_–CH_2_–O−); δ 4.33 (2H_DEAEMA_ −O–CH_2_–CH_2_–N–(CH_2_–CH_3_)_2_).

To quantify the w/w % of poly-DEAEMA in CDs-pDEAEMA, a known weight of CDs-pDEAEMA was weighed and dispersed in D_2_O and subsequently added with a known volume of acetonitrile (ACN) (used as an internal standard). The quantification was then obtained by using the following equation

$$mmol\mathrm{int}std÷\frac{\int H\mathrm{int}std}{n^{{}^{\circ}}H\mathrm{int}std}=mmol\;DEAEMA÷\frac{\int HDEAEMA}{n^{{}^{\circ}}HDEAEMA}$$

### Chemical–Physical Characterization

#### Size Exclusion Chromatography

Weight-average molecular weight (*M*_w_), number-average molecular weight (*M*_n_), and polydispersity (PD) of the poly-2-(diethylamino)­ethyl methacrylate chains bonded to the CDs surface were determined by size exclusion chromatography (SEC). To do so, 20 mg of CDs-pDEAEMA was first dispersed in 1 M HCl (2 mL) and kept under stirring for 15 h at 80 °C, achieving the detachment of the polymer chains from the CDs surface.

SEC analysis of the treated sample was thus performed using a Yarra 3 μm SEC—2000 column connected to an Agilent 1260 Infinity II Multi-Detector GPC/SEC system (Milan, Italy). The chromatography was carried out at 30 °C in citrate-phosphate buffer 0.15 M pH 5 as the mobile phase with a flow of 0.8 mL min^–1^, operating column calibration with PEO standards (ReadyCal-Kit PEO/PEG, Merck, Italy).

#### Atomic Force Microscopy

The dimensional characterization of CDs-pDEAEMA was assessed by atomic force microscopy (AFM) using a Bruker FAST-SCAN microscope equipped with a closed-loop scanner (maximum scanning areas: 35 μm × 35 μm in *X* and *Y*, and 3 μm in *Z*). Scans were acquired with a FAST-SCAN-A probe having a tip radius of 5 nm. The sample was prepared by dispersing 0.1 mg mL^–1^ CDs-pDEAEMA in ultrapure water, depositing 15 μL of the dispersion onto a mica substrate, and drying it gently under reduced pressure prior to analysis.

#### UV and Fluorescence Spectroscopy

The optical absorption and emission profiles of naked CDs and CDs-pDEAEMA were evaluated by UV and fluorescence spectroscopy using a Shimadzu UV-2401PC dual-beam spectrophotometer and a Jasco FP-8500 spectrofluorometer, respectively. The samples were dispersed in ultrapure water at the concentration of 0.025 mg mL^–1^ and analyzed by measuring the absorption spectra within the 200–800 nm range as well as recording three-dimensional emission spectra 350 to 800 nm setting excitation wavelengths ranging from 320 to 700 nm and a 10 nm acquisition interval.

### Complexation Studies

Complexation studies were carried out by both a gel retard assay and dynamic light scattering (DLS) measurements. Before analysis, the complexes were prepared in nuclease-free HEPES-glucose buffer at pH 7.4 (HGB: HEPES 10 mM, glucose 5% w/v) by incubating a fixed amount of scrambled siRNA with rising concentrations of CDs-pDEAEMA. In detail, 10 μL of siRNA was mixed with 10 μL of CDs-pDEAEMA dispersions, each time at increasing concentration, by gently pipetting and incubating 30 min at room temperature. The so obtained complexes present a final siRNA concentration of 0.1 mg mL^–1^ and CDs-pDEAEMA/siRNA w/w ratios (*R*) equal to 1, 3, 5, 7.5, 10, 15, 20.

Retardation assay was thus performed by loading 10 μL of each sample onto a 1.5% agarose gel prepared by heat-assisted dispersion of 750 mg of agarose in 50 mL of tris acetate/EDTA buffer (TAE) pH 8. The electrophoresis was thus carried out in the same buffer, setting the power supply to 100 V for 20 min. The gels were visualized by means of a UV transilluminator and captured with a digital camera.

For the DLS monitoring of the complexation process, 10 μL of complexes obtained as described above was first diluted with 60 μL of HEPES 10 mM and then analyzed to evaluate the hydrodynamic diameters and size distribution. The mean hydrodynamic diameters were measured using a Malvern Zetasizer NanoZS instrument equipped with a 632 nm laser and with a fixed scattering angle of 173°. The *Z*-average and PDI values were obtained from analysis of the correlograms. The examination of the changes in surface charge were carried out after further dilution with HEPES 10 mM (1:10 v/v) via aqueous electrophoresis measurements at 25 °C, employing the same instrument. Zeta-potential values (mV) were thus calculated from the electrophoretic mobility using the Smoluchowski relationship.

### Stability Studies

The stability of the complexes against polyanionic exchange was evaluated in the presence of human serum albumin. The complexes, prepared as described earlier for the electrophoretic assay, were incubated at room temperature for 4 or 8 h with an albumin dispersion in HGB to obtain a final concentration of polyanionic protein equal to 45 mg mL^–1^. Stability was determined by tracking their electrophoretic mobility over time and comparing it to that of samples incubated without albumin.

A further stability study was conducted to analyze if CDs-pDEAEMA can protect siRNA against endonucleases. To do so, complexes were prepared by gently pipetting the same volumes (5 μL) of CDs-pDEAEMA-scrambled siRNA and dispersions in HGB and incubated 30 min at room temperature to obtain the w/w ratios of 0 (naked siRNA), 3, 4, 5, 7.5. The samples were divided into 2 aliquots and maintained for 1 h in orbital shaker (37 °C) in the absence or presence of 2 μL of RNAse A (mol/mol ratio siRNA/RNAse A: 50). After this time, both sets of samples were incubated at 37 °C with 2% SDS (5 μL, 10 min) to achieve RNAse A inactivation and then with heparin (4 μL, 1000 U mL^–1^, 20 min) to pursue exhaustive polyanionic exchange. Stability was assessed by monitoring their electrophoretic mobility over time or directly quantifying residual RNA and comparing it to samples incubated in the absence of RNAse A.

To quantitatively determine the degradation of the oligonucleotides in the complexes, both densitometry on the electrophoresis gels’ pictures acquired by a digital camera using ImageJ software as well as the quantification of siRNA by RediPlate 96 RiboGreen RNA Kit were performed. Data were expressed as a percentage relative to the RNAse A free samples.

### In Vitro Studies

#### Erythrocompatibility

Healthy erythrocytes were isolated from 4 mL of freshly collected venous blood by centrifugation at 500*g* for 10 min. The resulting pellet was washed four times with PBS at pH 7.4 until the supernatant was clear and colorless. The washed erythrocytes were resuspended to a final volume of 4 mL and diluted 50-fold with PBS. Subsequently, 1.425 mL of the diluted erythrocyte suspension was mixed with 75 μL of siRNA complexes (CDs-pDEAEMA/siRNA) dispersions in HEPES 10 mM obtained at the different w/w ratios (CDs-pDEAEMA/siRNA: 3, 4, 5 and 7.5) or with the same volume of CDs-pDEAEMA at equivalent concentrations of complexing agent. The mixtures were kept at 37 °C under orbital shaking for 1 h and then centrifuged again at 500*g* for 10 min. Hemoglobin release, indicating the degree of erythrolysis, was measured spectrophotometrically at 570 nm. Results were normalized against the absorbance registered after incubation of erythrocytes with TRITON X-100, used as a positive control.

#### Cell Culture

MDA-MB-231 breast cancer cells and human dermal fibroblasts (HDF) were purchased from Merck (Italy). The cells were cultured in Dulbecco’s Modified Eagle Medium (DMEM) supplemented with 10% fetal bovine serum (FBS, Euroclone), 1% penicillin/streptomycin (1000 U mL^–1^ penicillin and 10 mg mL^–1^ streptomycin, Euroclone), and 1% l-glutamine (Euroclone) under a humidified atmosphere containing 5% CO_2_ at 37 °C. Opti-MEM I Reduced Serum Medium was purchased from Thermo Fisher and used for transfection studies. For the in vitro studies, all samples were filtered through 0.22 μm sterile syringe filters before testing.

#### Cytocompatibility

To test the cytocompatibility of the CDs-pDEAEMA and the complexes obtained with non-targeting siRNA sequence (scrambled siRNA), MTS assay was carried out on the triple-negative breast cancer cell line MDA-MB-231 and on human dermal fibroblasts (HDF). Cells were plated in a 48-well plate at a density of 4 × 10^4^ cells per well and cultured with 400 μL of supplemented DMEM for 24 h to let them adhere. On completion, the medium was discarded, the wells were washed 2 times with sterile DPBS, and the cells were incubated with dispersions of Lipofectamine/scrambled siRNA, formulated as described by the manufacturer, CDs-pDEAEMA/scrambled siRNA (Thermo Fischer) complexes at w/w ratios of 0 (naked scrambled siRNA), 3, 4, 5, 7.5 or equivalent concentrations of bare CDs-PDEAEMA obtained in Opti-MEM (Thermo Fisher) medium. The complexes’ dispersions in transfection medium were prepared in order to obtain the final siRNA concentration in each well equal to 100 nM. After 48 or 72 h, the wells were washed twice with DBPS and 240 μL of MTS diluted in complete DMEM (1:6 dilution) was added to each well and maintained at 37 °C for 1 h. Cell viability was determined by measuring absorbance at 492 nm using an Eppendorf Plate Reader and expressed as a percentage relative to untreated control cells cultured in OPTI-MEM alone.

#### Cytotoxicity of the Therapeutic Complexes and Survivin-Silencing Tests

The efficacy of the therapeutic complexes was evaluated with both cytotoxicity and ELISA studies. The assessment of possible reduction in cell viability after incubation with complexes formulated with BIRC-5-silencing siRNA was verified through the MTS assay on HDF and MDA-MB-231 cell lines. The protocols for seeding, sample incubation, the MTS colorimetric assay, and viability measurements were replicated from the cytocompatibility tests. However, this time, the cell viability was investigated after incubation with complexes produced using BIRC5-silencing siRNA instead of scrambled siRNA, at w/w ratios CDs-pDEAEMA/silencer BIRC5 of 3 and 4 and compared to naked BIRC5 siRNA (w/w ratio: 0) and with a positive transfection control complex consisting of silencer BIRC5 and Lipofectamine formulated as described by the manufacturer. To evaluate the differences in the expression of the survivin protein as a consequence of BIRC-5 silencing, the same samples were tested on MDA MB-231, cultured, and seeded as described above. The cells were lysed with 0.15 mL of TRITON-X 100 lysis buffer per well, maintaining plates in an ice bath. The resulting lysates were collected, centrifuged at 14,000 rpm for 15 min, and the quantification of survivin protein was carried out via a Human survivin ELISA kit (antibodies.com) in accordance with the manufacturer instructions. 25 μL of each lysate was used to determine the total protein content of each sample via a BCA assay, following the manufacturer’s protocol. Data were expressed as picograms of survivin per microgram of total proteins and then normalized with respect to the untreated control, considered as 100% of survivin expression.

#### Cell Uptake Studies

The qualitative cellular uptake of CDs-pDEAEMA complexes was investigated on MDA-MB-231 and HDF cell lines. Cells were seeded on 8-well plates (Glass Coverslips) at a density of 1 × 10^4^ cells per chamber and maintained to adhere in complete DMEM at 37 °C in a humidified environment for 24 h. Later, the medium was discarded, the wells washed twice with sterile DPBS, and fresh Opti-MEM containing CDs-pDEAEMA/siRNA Cy5 (*R*4), lipofectamine/siRNA Cy5, naked siRNA Cy5, or medium alone were incubated for 4 and 24 h. Following the incubation period, the chamber contents were removed, the cells were fixed with a 4% formaldehyde solution in the same buffer (10 min, RT), and then the nuclei were stained with 4′,6-diamidino-2-phenylindole (DAPI) (10 min, RT). The wells were washed three times with DPBS both before and after fixation. Uptake micrographs were captured using a Zeiss fluorescence microscope, with images recorded by an Axio Cam MRm camera and a 100× magnification immersion objective using DAPI (excitation: 359 nm, emission: 457 nm), Texas Red (excitation: 561 nm, emission: 594 nm), and Cy5 (excitation: 649 nm, emission: 670 nm) channels was used for all treated cell lines and experiments, ensuring consistent exposure times.

Quantitative analysis of siRNA uptake was also conducted. Specifically, MDA-MB-231 and HDF cells were seeded in a 48-well plate at a density of 60 × 10^4^ cells per well and incubated with Opti-MEM dispersions of CDs-pDEAEMA/scrambled siRNA (not labelled), CDs-pDEAEMA/siRNA Cy5 (*R*4), lipofectamine/siRNA Cy5, naked siRNA Cy5, or OPTI-MEM alone. The plates were maintained under standard conditions (37 °C, 5% CO_2_) for 4 and 24 h. At the end of the incubation, the medium was removed, and the wells were washed three times with DPBS. The cells were then lysed using 0.15 mL of TRITON-X 100 lysis buffer per well, maintaining plates in an ice bath. The lysates were collected, centrifuged at 14,000 rpm for 15 min, and the supernatant was divided into two aliquots. 25 μL was used to determine the total protein content of each sample via a BCA assay, following the manufacturer’s protocol, while 100 μL was analyzed through a Jasco FP-8500 spectrofluorometer measuring the fluorescence intensity using Cy5 excitation and emission wavelengths. Fluorescence readings were normalized to the total protein content.

#### Statistical Analysis

All experiments were conducted in three or six replicates. Data are presented as mean values ± standard deviation. Statistical significance was assessed using one-way analysis of variance (ANOVA), with significance levels set at *p < 0.05, **p < 0.01, and ***p < 0.001 unless otherwise specified.

## Results and Discussion

### Synthesis and Chemical–Physical Characterization of CDs-pDEAEMA

This work aimed to develop a single nanoplatform capable of providing gene silencing of anti-apoptotic proteins in TNBC, alongside the ability to monitor therapy progression via CDs based imaging. In this regard, a novel protocol has recently been developed to enable the scalable synthesis of N,S-doped CDs with uniform size distribution (*d*: 5.2 ± 0.3 nm), multicolor emission, efficient NIR photothermal conversion, and high surface area.^28^ These zero-dimensional nanoparticles have already demonstrated capable of photo-sensitive drug delivery and selective nanotoxicity against breast cancer cells by enhancing reactive oxygen species production.^29,30^ Grounded on their multifunctionality, these CDs were selected as a multifunctional core to be passivated on the surface in order to achieve customized biological features and physicochemical properties built for siRNA delivery for surviving silencing in breast cancer treatment. Survivin silencing was chosen since its overexpression is frequently found in TNBC and is associated with poor prognosis.^31,32^

To gain this tool, CDs were functionalized through atom transfer radical polymerization (ATRP), a controlled polymerization method particularly suitable for synthesizing polymers with well-defined structures that are essential for the development of pharmaceutical grade systems equipped with tailored functional properties. The surface functionalization of CDs was carried out through three synthetic steps without isolating the intermediates and exploiting surface carboxylic groups amenable to further coupling reactions (Scheme 1). Before functionalization with the BIBB initiator, a spacer carrying a reactive hydroxyl end-chain was first grafted. Despite the confirmed presence of hydroxyl groups on the surface of the selected CDs, which may allow the production of the macroinitiator CDs-BIB, attempts to directly grow the polymer chain on the surface of the CDs showed unsatisfactory results. For this reason, the first step involved an amide coupling reaction with ethanolamine on the surface of the CDs carried out in DMF, exploiting the carboxyl groups that were preactivated via carbodiimide (DCC/NHS). During the second step, hydroxyl functions of ethanolamine were used to link the initiator BIBB to the spacer, obtaining the macroinitiator CDs-ethanolamine-BIB. Finally, the third step consisted of the ATRP of the cationic 2-(diethylamino)­ethyl methacrylate (DEAEMA) monomers to achieve the chain growth of positively charged moieties onto the CDs surface, able to complex through ionic interactions the nucleic acids. To do so, previously degassed methanol was first added to obtain a 50:50 DMF/methanol mixture 50:50 v/v. Given the effect of polar solvents on ATRP polymerizations, this change in the reaction conditions was made aiming to promote rapid polymerization while preserving control over the reaction.^33^ Polymerization was thus carried out by adding CuBr as catalyst and Bpy as complexing ligand and maintaining the reaction flask under an argon atmosphere for 24 h at 50 °C. The crude reaction was then double purified by dialysis and column SEC to give a brownish solid after freeze-drying, henceforth named CDs-pDEAEMA. CDs-pDEAEMA were characterized using both ^1^H NMR and FT-IR spectroscopy. In the ^1^H NMR spectrum of the purified CDs-pDEAEMA conjugate (Figure 1), there were characteristic resonances of DEAEMA protons at 1–4.5 ppm, while no peaks corresponding to unreacted monomers were detected. Indeed, there were no detectable resonances at 5.5–6.5 ppm, which are typical of acrylic double bonds. Moreover, this analysis enabled the quantification of the w w^–1^ percentage of poly-DEAEMA in the CDs-pDEAEMA sample using an internal standard (Figure S1), which was determined to be 78%. The ^1^H NMR data were employed to estimate the number of monomers constituting the polymer chain by comparing the integrals of the two methyl groups of poly-DEAEMA (δ 1.28) with a methylene group attributable to ethanolamine (δ 3.73), used as a reference. From these calculations, the average degree of polymerization ( ${\overset{®}{DP}}_{n}$ ) of DEAEMA monomers was determined to be 30 ± 2.

**Scheme 1:**
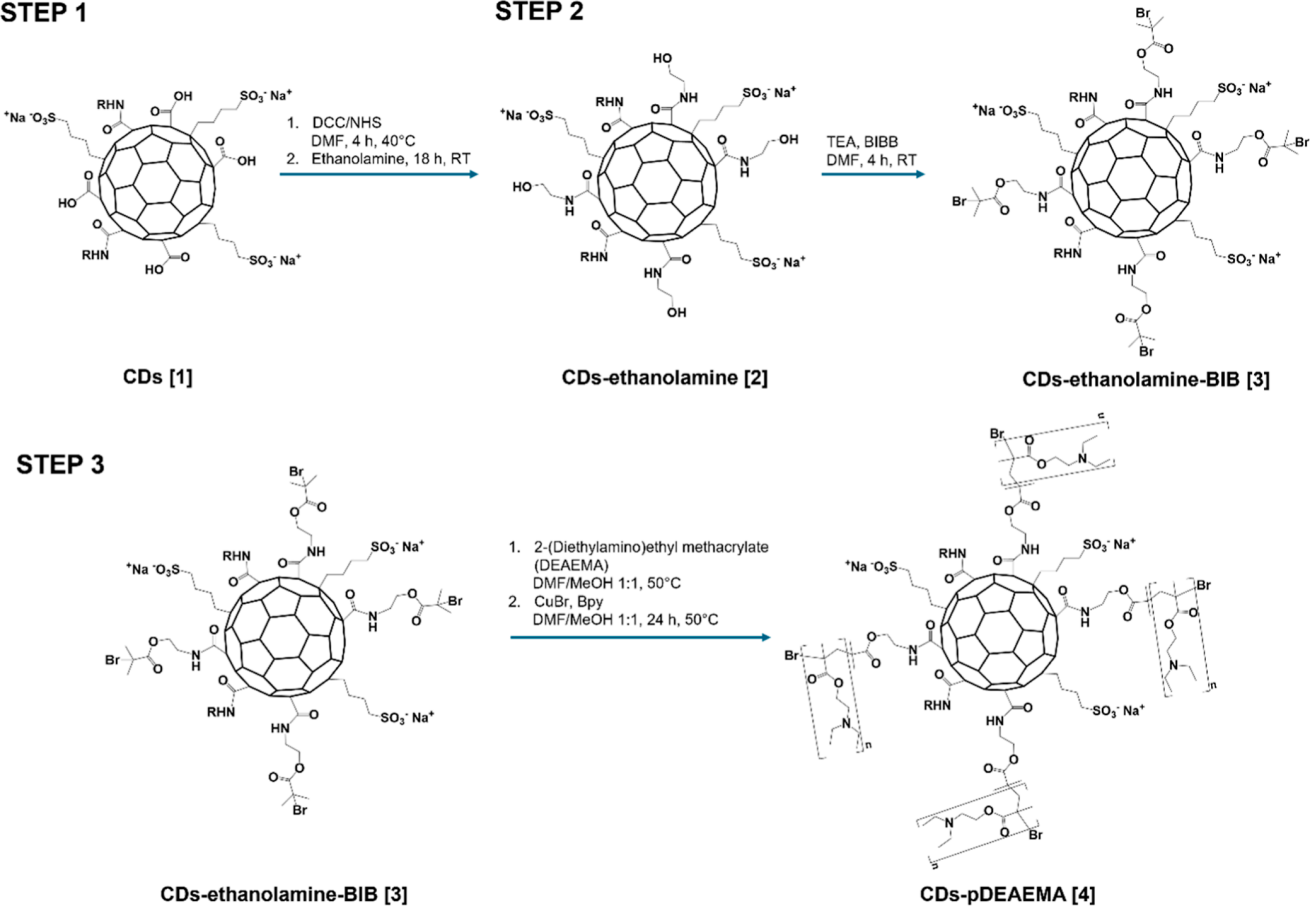
Synthetic Pathway for the CDs-pDEAEMA Conjugate. Synthetic Steps without Isolation of Intermediates to Achieve Surface Functionalization of CDs via (Step 1) Addition of Ethanolamine Spacer (CDs-Ethanolamine), Followed by (Step 2) Formation of the Macroinitiator (CDs-Ethanolamine-BIB) and Subsequent ATRP Polymerization of DEAEMA Cationic Monomers (CDs-pDEAEMA)

**Figure 1:**
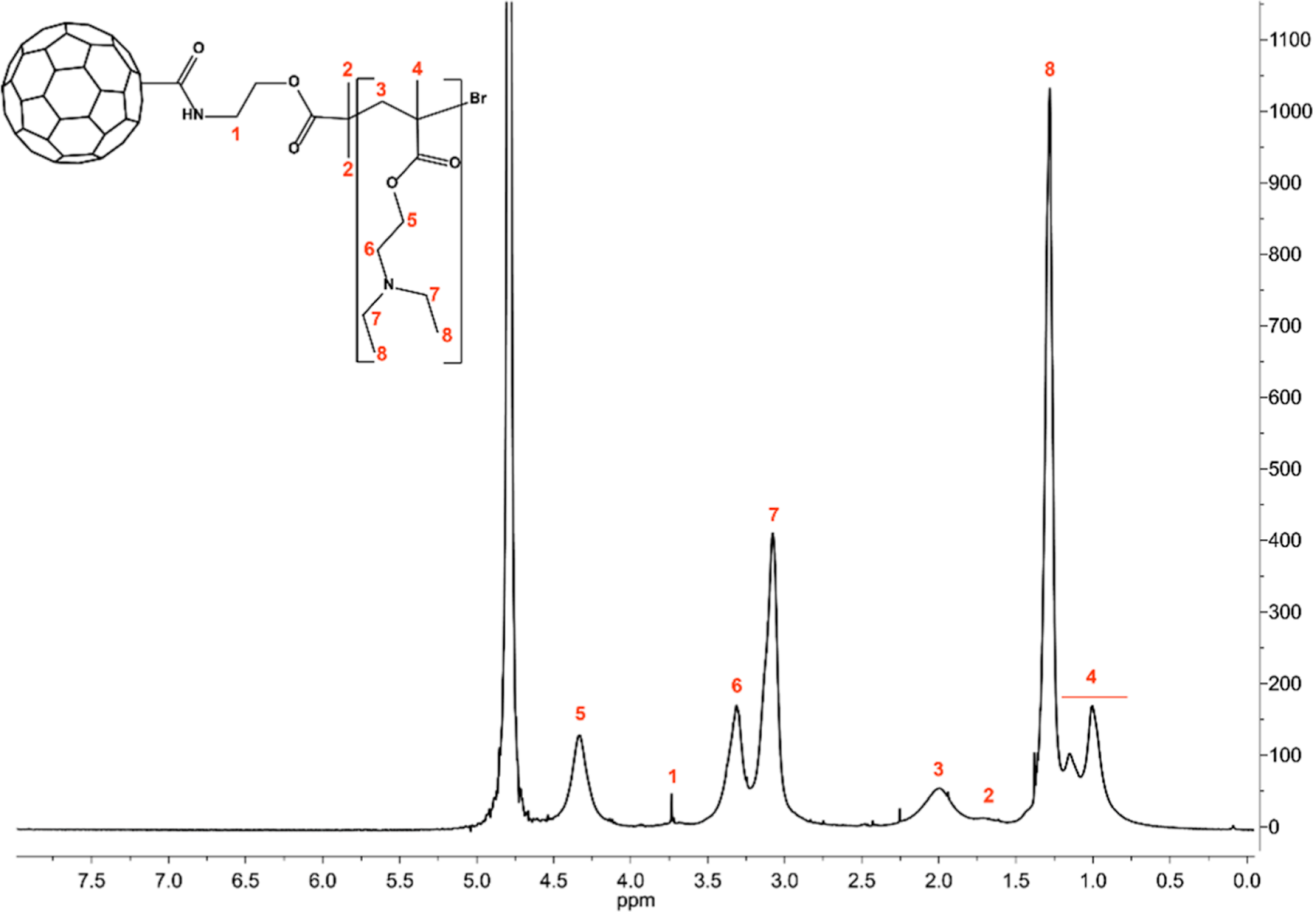
^1^H NMR spectroscopy of the derivative CDs-pDEAEMA, D_2_O, 400 MHz.

To gain more accurate and reliable results, the molecular weight of the polymer chain was also determined through SEC analysis carried out after the acid-catalyzed hydrolysis of CDs-pDEAEMA. SEC chromatography of the polymer chain showed ${\mathrm{M̅}}_{w}$ = 8100, *M*_p_ = 6000, and ${\mathrm{M̅}}_{n}$ = 5400, with a polydispersity of 1.68. These data are in accordance with NMR results, as we can calculate from the ${\mathrm{M̅}}_{n}$ a ${\overset{®}{DP}}_{n}$ of 28 DEAEMA monomers, corroborating the previous study. Furthermore, CDs-pDEAEMA were also analyzed by using FT-IR spectroscopy (Figure 2a) and compared to bare CDs. While the infrared spectrum of CDs exhibited bands attributed to the presence of carboxylic acids (stretching, ν C=O, 1715 cm^–1^), amide (ν C=O, 1640 cm^–1^), hydroxyl (ν O–H, 3420 cm^–1^), and amine (ν N–H, 3420 cm^–1^) functional groups, that are in full agreement with surface analyses reported in the literature,^28^ the spectrum of CDs-pDEAEMA confirmed the successful polymerization through an increase in the intensity of bands corresponding to the stretching of C–H (2967 cm^–1^), of C–N (1377 cm^–1^), and of C–O–C (1150 cm^–1^), indicating a rise in alkyl chains, amines, and esters, consistent with the presence of poly-DEAEMA chains.

**Figure 2:**
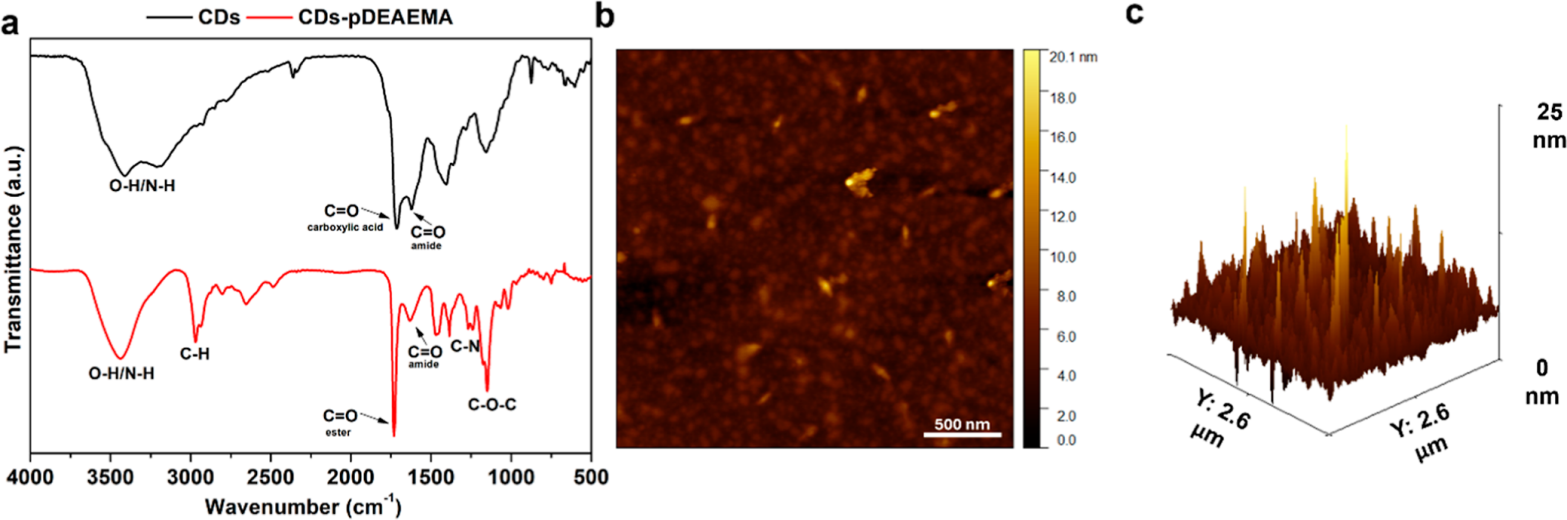
Characterization of CDs-pDEAEMA. (a) FT-IR spectra of native (black) and functionalized (red) CDs. (b)­AFM analysis of CDs-pDEAEMA and 3D view of AFM analysis of CDs-pDEAEMA.

Furthermore, the presence of esters was supported by the peak assigned to the stretching of ester C=O (1730 cm^–1^), which appeared to be shifted compared to the carboxyl C=O band in the spectrum of bare CDs.

The size distribution of CDs-pDEAEMA was assessed using atomic force microscopy (Figure 2b,c), revealing an average dimension of 12.3 ± 1.4 nm. These measurements agree with the presence of a polymeric shell coating, which increased the average diameter of the native CDs by 7.1 nm. This suggests that each pDEAEMA chain has a hydrodynamic diameter of 3.55 nm. This increase suggests the potential to evade renal filtration, possibly extending the circulation time of the delivery system.^34^ However, after proper biodistribution and cleavage of the ester bonds between pDEAEMA and CDs by plasma and cellular esterases (particularly under the acidic conditions present in the tumor microenvironment), both components can, in principle, be eliminated via renal clearance (with a renal cutoff of 5.5 nm).^35^ The physicochemical properties were further evaluated through Zeta-potential analysis, which recorded a positive surface charge of +33.3 ± 6.58, indicating a good stability against aggregation and, most importantly, an optimal charge for effective siRNA complexation.

### Optical Properties of CDs-pDEAEMA

The capability of CDs of acting as fluorescence imaging agents is widely recognized in literature, as well as the possibilities to tune their optical properties by carefully modifying their surface with chosen passivating agents.^26,27,36^ To assess how the presence of the polymeric shell may have influenced these properties, the absorbance and emission spectra of both native and functionalized CDs were compared (Figure 3). The absorption spectrum of CDs-pDEAEMA (Figure 3a) revealed the most significant differences in the range of 300–450 nm, where a broadening of the highest peak of the starting CDs was observed, along with a slight red shift of the λ max from 343 to 348 nm. Additionally, a new shoulder appeared at 400 nm, while the spectra remained quite similar between 450 and 700 nm, except for a mild increase in absorbance at the 475 nm shoulder and a red shift of the peak at longer wavelengths (from 557 to 565 nm).

**Figure 3:**
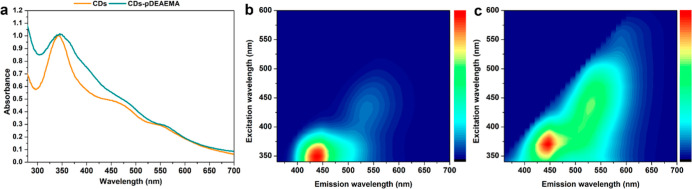
Optical characterization of CDs-pDEAEMA. (a) UV spectra of native CDs (orange) and CDs-pDEAEMA (green); 3D fluorescence spectrum of CDs (b) and CDs-pDEAEMA (c).

From the fluorescence emission standpoint, the functionalization of CDs with pDEAEMA led to a general broadening of the emission spectrum, resulting in more intense fluorescence in the orange-red region compared with the bare CDs (Figure 3b,c). Specifically, the maximum emission of CDs-pDEAEMA (Figure 3c) was observed upon excitation at 370 nm rather than at 350 nm as in the case of bare CDs (Figure 3b). Moreover, the polymeric coating seemed to enhance the emission in the range of 475–630 nm, perhaps due to electron surface trap effects due to changes in surface dipole.^37,38^ This result is particularly significant, as emission in the red region would enable deeper tissue penetration and improved resolution for enhanced theranostic performance.^39,40^ Additionally, a comparison of the two spectra shows that CDs-pDEAEMA emit even when excited at wavelengths above 520 nm, an effect not observed in the original CDs, further supporting their potential as superior bioimaging tools for more precise visualization with minimal interference.

### Complexation Studies by Electrophoresis and Dynamic Light Scattering

The ability of CDs-pDEAEMA to form interpolyelectrolytic complexes with siRNA was studied through agarose gel electrophoresis and monitored through size and Zeta-potential measurements. Dispersions of increasing amounts CDs-pDEAEMA and fixed concentration of non-targeting (scrambled) siRNA, used as a model, were mixed in RNAse-free medium and incubated to achieve various CDs-pDEAEMA/siRNA weight ratios (*R*) ranging from 1 to 20. Migration of uncomplexed siRNA was evaluated by electrophoresis. As shown in Figure 4a, CDs-pDEAEMA effectively bound siRNA sequences starting from *R*3 (corresponding to an N/P ratio equal to 3.8), with a minimum weight ratio resulting in the interruption of electrophoretic migration, thus indicating neutralization of nucleic acid strand charges and complex formation. To more precisely track the establishment of the complexes, parallel studies were conducted to examine changes in the average hydrodynamic diameter and surface charge after interaction of CDs-pDEAEMA with nucleic acids. These studies confirmed the electrophoresis data, indicating complex formation starting at a w w^–1^ ratio of 3. Specifically, as shown in Figure 4b, the lowest tested ratio (*R*1) does not allow for the neutralization of the Zeta-potential, meaning that the positive charges of CDs-pDEAEMA were insufficient to fully interact with all nucleic acid chains. Also the *Z*-average values obtained at *R*1 suggest a partial complexation of siRNA, showing nanoparticles of about 300 nm (Figure 4b). At a ratio of *R*3, a quasi-neutral Zeta-potential is observed together with a first decreasing in hydrodynamic diameter (∼240 nm), possibly depicting a complex undergoing dynamic conformational changes, where interactions are not yet fully stabilized. At ratios of 4 and 5, the optimal conditions were reached, with neutral Zeta-potential and the formation of collapsed structures of roughly 200 nm, that appeared to plateau up to *R*20. However, a sharp increase in Zeta-potential values is clearly appreciated at *R* > 7.5, reaching a maximum of +33.3 ± 6.58 mV at *R*20. This trend implies that the arrangement of fully stable siRNA/CDs-pDEAEMA interpolyelectrolytic complexes are obtained at *R* 4–7.5. After that a layer-by-layer adsorption of cationic components can be obtained using excess CDs-pDEAEMA. Beyond that point (*R*: 10, 15, 20), a layer-by-layer adsorption of cationic components can occur when using excess CDs-pDEAEMA. Given these data, the complexes with ratios between 3 and 7.5 were considered the better candidates for further studies.

**Figure 4:**
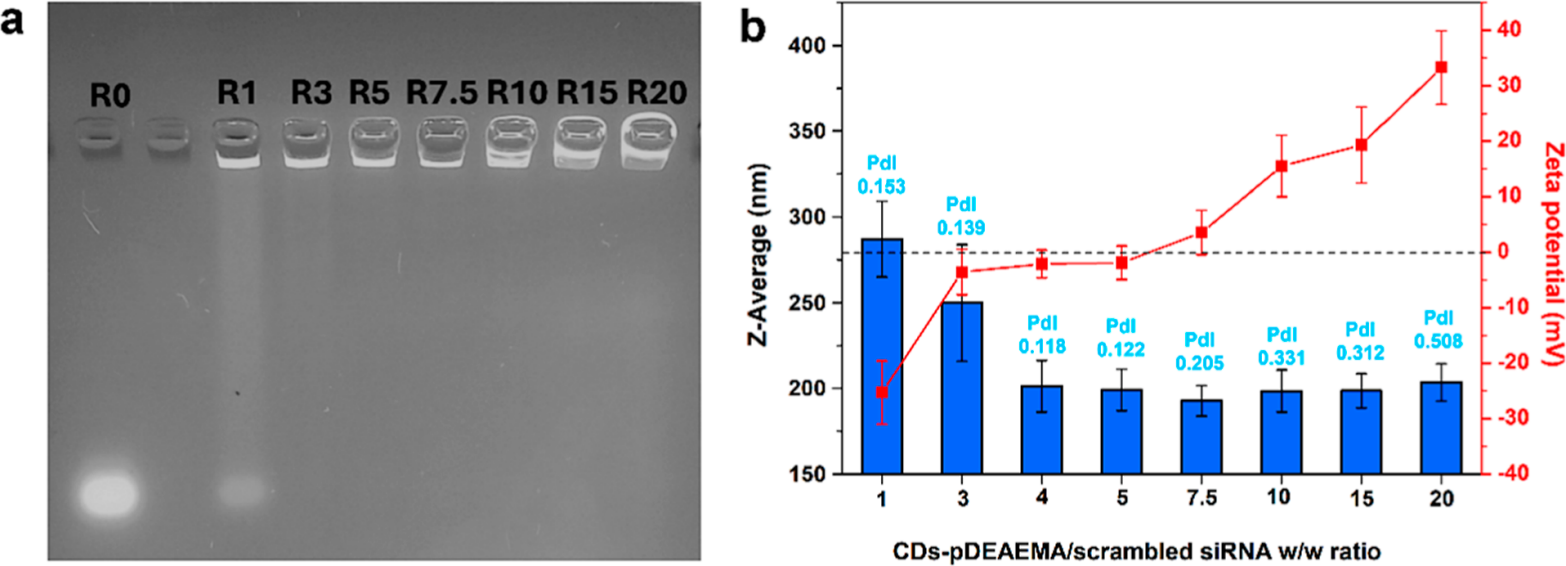
Analyses of complexes formation testing different CDs-pDEAEMA/siRNA weight ratios (*R*) carried out by (a) electrophoretic migration and (b) hydrodynamic diameters, Zeta potential, size distribution measurements.

Since the intravenous route is one of the most common administration pathways for siRNA therapy, it was deemed necessary to assess the stability of the complexes against polyanionic exchange, a phenomenon that could lead to electrostatic competition and, in some cases, the disassembling of the complex.^41^ Therefore, CDs-pDEAEMA/siRNA complexes were incubated in the presence of albumin, the most abundant polyanionic protein in the bloodstream, for 4 or 8 h. Considering that polyanionic exchange may necessitate a higher amount of complexing agent to ensure complex stability, although previous studies had identified the most promising ratios (*R* 3, 4, 5, 7.5), higher ratios (*R* 10, 15) were also tested in this study. Interestingly, as shown in Figure 5a, all the tested ratios ensured stability in the presence of albumin at both analyzed time points, except for a slight destabilization observed for *R*3 after 8 h, incubation time tested to expose the delivery systems to stress conditions.

**Figure 5:**
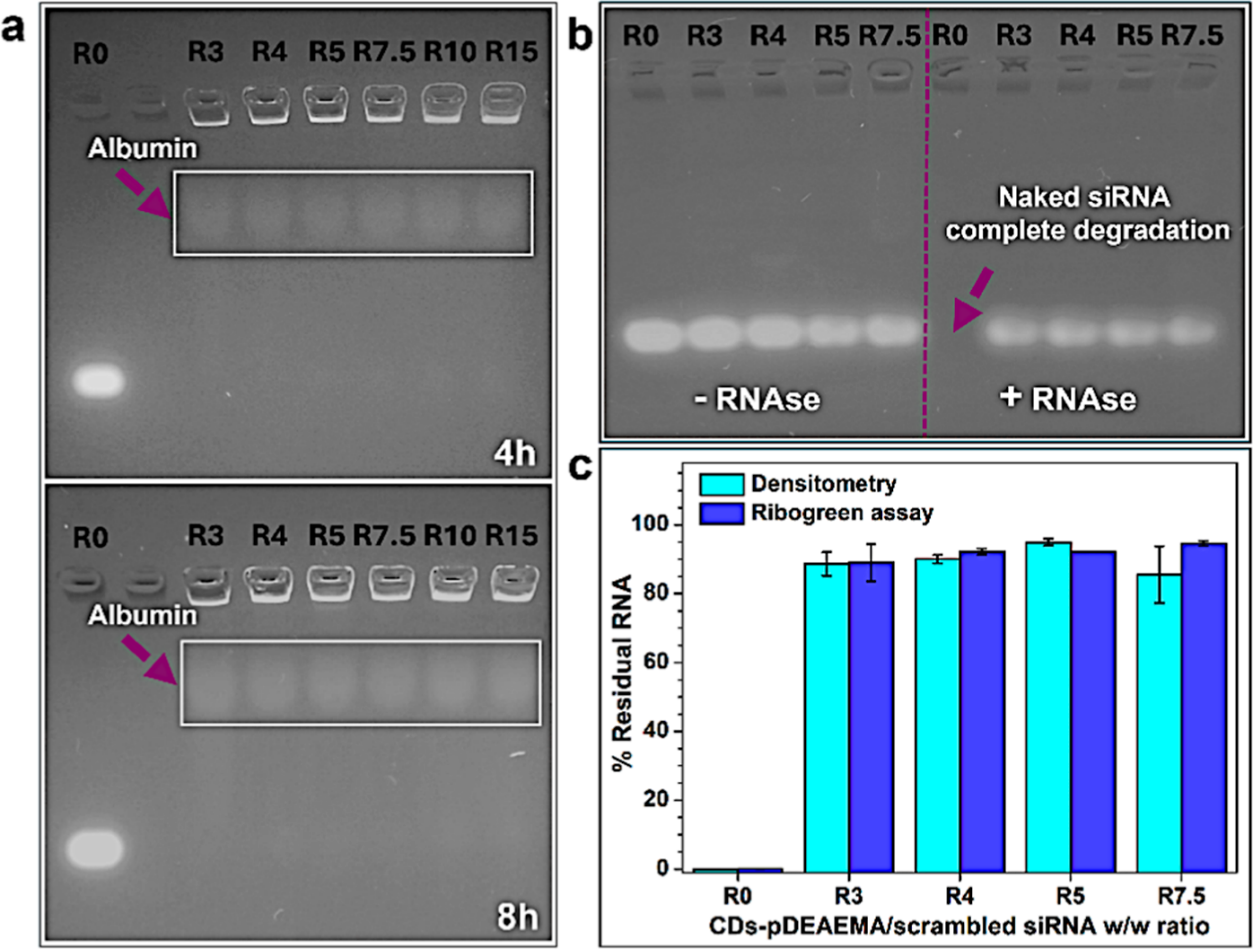
Stability studies of CDs-pDEAEMA/scrambled siRNA. (a) Stability against polyanionic exchange monitored through electrophoretic migration of complexes after 4 h or 8 h incubation with albumin; stability of complexed gene material against RNAse A (b) assessed through electrophoretic migration and (c) residual RNA percentages quantified by densitometry and RiboGreen assay.

Having confirmed the stability of the complexes against polyanion exchange, the protective effect of CDs-pDEAEMA toward degradation of the transported genetic material by circulating endonucleases was investigated. To achieve this, the complexes formed at selected ratios (*R*: 3, 4, 5, 7.5) were tested via electrophoretic analysis after a 1 h incubation with RNAse A. This assay evaluates the fraction of nucleic acids that remain intact following endonuclease exposure. To accurately visualize the residual non degraded siRNA, the complete disassembly of the complex, achieved incubating with high concentrations of heparin, is required as a final step, allowing the RNA to migrate toward the positive pole during electrophoresis. In Figure 5b it is noticeable how the nucleic acid band in *R*0 ratio disappeared in the presence of RNAse, while it is still visible when siRNA is protected by CDs-pDEAEMA in the complexes at all the tested ratios. To understand the extent of this effect, residual RNA was quantified both by densitometry of the electrophoresis gel and by RibogGreen assay, a ready-to-use kit to quantify RNA. The plotted data showed that all complexes tested are able to preserve at least 85% (and up to 95%) of siRNA cargo against RNAse A degradation with consistent results regardless of the method of quantification. These findings demonstrate the strong overall stability of CDs-pDEAEMA/siRNA complexes, suggesting the possibility of delivering the intact therapeutic carrier to the target site after administration.

### Cell Compatibility

In view of possible administration, the first studies on cells were oriented to assess the compatibility of complexes obtained using scrambled siRNA with red blood cells. To have a clearer view, CDs-pDEAEMA were also tested alone at concentrations equivalent to the ones used to prepare complexes, with the aim to ensure that the delivery system can be considered completely safe. The studies conducted on red blood cells (Figure 6) showed that the complexes obtained with the lower ratios (*R*: 3, 4, 5) were entirely compatible, with erythrolysis levels comparable to those observed when incubating cells in phosphate buffer, values attributable to the stress endured by the cells during operational procedures (e.g., centrifugation, resuspension). Slightly higher values, though not concerning (as they remained around 10%), were recorded after incubation with the carrier CDs-pDEAEMA alone (without siRNA).

**Figure 6:**
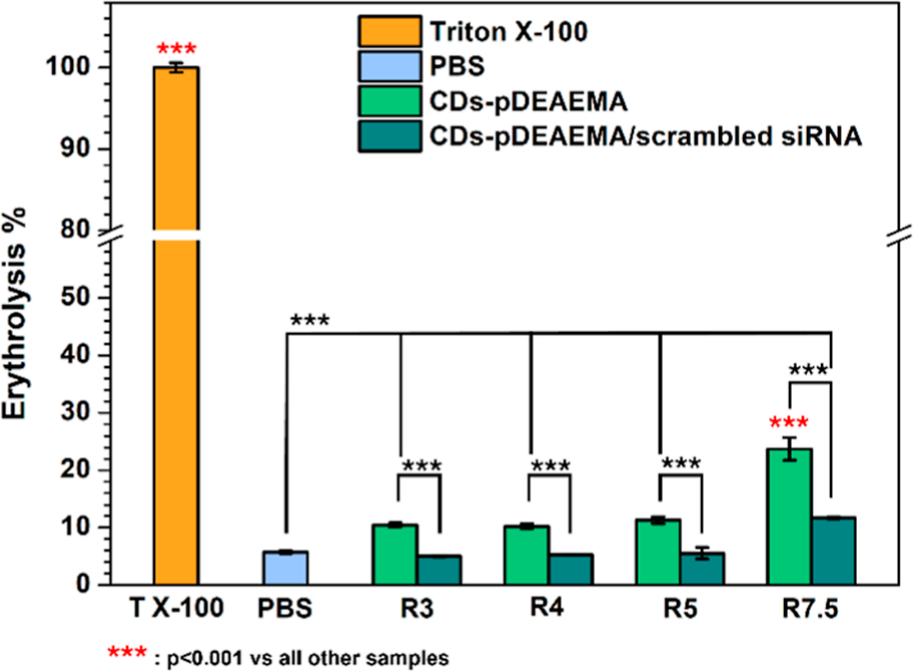
Compatibility with red blood cells. Studies conducted on CDs-pDEAEMA/scrambled siRNA complexes at w/w ratios of 3, 4, 5, and 7.5 (dark green), CDs-pDEAEMA alone at equivalent concentrations (light green), using PBS pH 7.4 (light blue) and Triton X-100 (orange) as negative and positive controls, respectively.

These results are not surprising, as it is well known that highly cationic polymers can cause damage to red blood cells.^42,43^ Nonetheless, considering the mild extent of lysis observed along with the high stability of the complexes demonstrated in previous studies, the collected data indicate that these systems are safe for red blood cells. However, different considerations apply to the highest tested ratio (7.5), where the toxicity was significantly higher, nearly doubling compared to the lower ratios.

Compatibility studies were then conducted on human dermal fibroblasts (HDF), used as models of healthy cells, as well as on the triple negative breast cancer cell line MDA-MB-231, to evaluate possible siRNA-independent “non-therapeutic” cytotoxic effects associated with the carrier. Hence, scrambled siRNA was also used in these studies, and once again, CDs-pDEAEMA were tested separately to evaluate any potential cytotoxicity of the empty vehicle. The entry *R*0 represents cell medium or scrambled siRNA, used as controls of empty vehicle or scrambled siRNA complexes, respectively. Furthermore, the complex obtained with Lipofectamine as transfection reagent was assayed for comparative purposes. As shown in Figure 7a,b, the results on HDF closely mirror those observed in red blood cell studies, confirming full compatibility for complexes formed at lower ratios (3, 4, 5) as well as for equivalent concentrations of the empty carrier. Similarly, a reduction in cell viability was noted for the highest ratio (7.5) tested at both time points, with viability dropping to approximately 70% after 48 h and further declining to 65% after 72 h, falling below the cytocompatibility threshold.^44^ Finally, lipofectamine complexes followed a similar trend, resulting in being cytocompatible after 48 h, with registered viability of about 85%, while showing a slight toxicity after 72 h (about 60% viability). These data are in accordance with previous literature that reports a decrease in cell viability after treatment with lipofectamine complexes, due to its cationic nature.^45^

**Figure 7:**
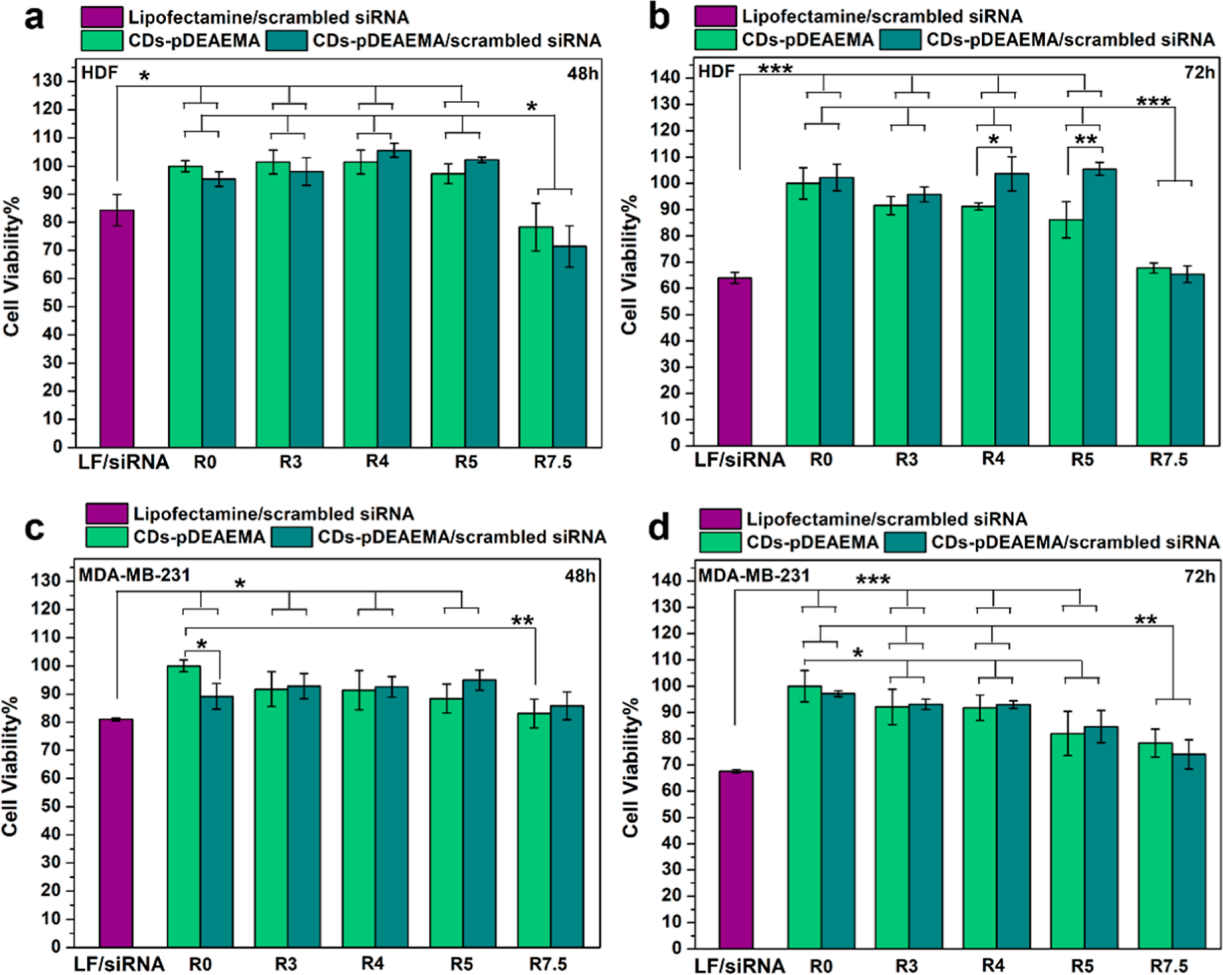
Cell compatibility studies on HDF (a,b) and MDA-MB-231 (c,d) conducted on CDs-pDEAEMA/scrambled siRNA complexes at w/w ratios of 3, 4, 5, and 7.5 (dark green), CDs-pDEAEMA alone at equivalent concentrations (light green), lipofectamine/scrambled siRNA (purple) used as positive transfection control and compared with controls incubated with cell medium (*R*0, light green) or naked scrambled siRNA (*R*0, dark green).

The MDA-MB-231 cell line (Figure 7c,d) exhibited greater resistance against the highest ratio tested, while, once again, lipofectamine complexes seemed to exert a mild cytotoxic effect after 72 h incubation. Nonetheless, the overall trend remained consistent with that observed in HDF, demonstrating that ratios 3, 4, and 5 are non-toxic on both cell lines. On the other hand, compatibility data from red blood cells, HDF, and MDA-MB-231 indicated a mild toxicity of the highest ratio leading to the exclusion of *R* 7.5 from further studies. More specifically, despite the comparable results among *R*3, *R*4, and *R*5, only the two lowest ratios were selected for subsequent experiments involving therapeutic siRNA, ensuring that the most promising systems with minimal cationic complexing agent content were tested.

### Efficacy of Survivin-Silencing siRNA Complexes

To evaluate whether CDs-pDEAEMA can deliver therapeutic BIRC5 silencer siRNA and assess the impact of transfection on tumor cell growth, the effect of CDs-pDEAEMA/BIRC5 siRNA on the viability of MDA-MB-231 and HDF cells was examined after 48 and 72 h of incubation with the therapeutic carriers.

Figure 8 illustrates that the *R*3 and *R*4 complexes effectively reduced MDA-MB-231 cell viability (Figure 8a) through siRNA therapy as early as 48 h. The effect was more pronounced at the lower ratio, achieving approximately a 40% reduction in viability, which remained consistent up to 72 h of treatment. Notably, the Lipofectamine complex exhibited a weaker reduction in viability when accounting for carrier-induced cytotoxicity, yielding results comparable to those observed with scrambled siRNA (Figure 7).

**Figure 8:**
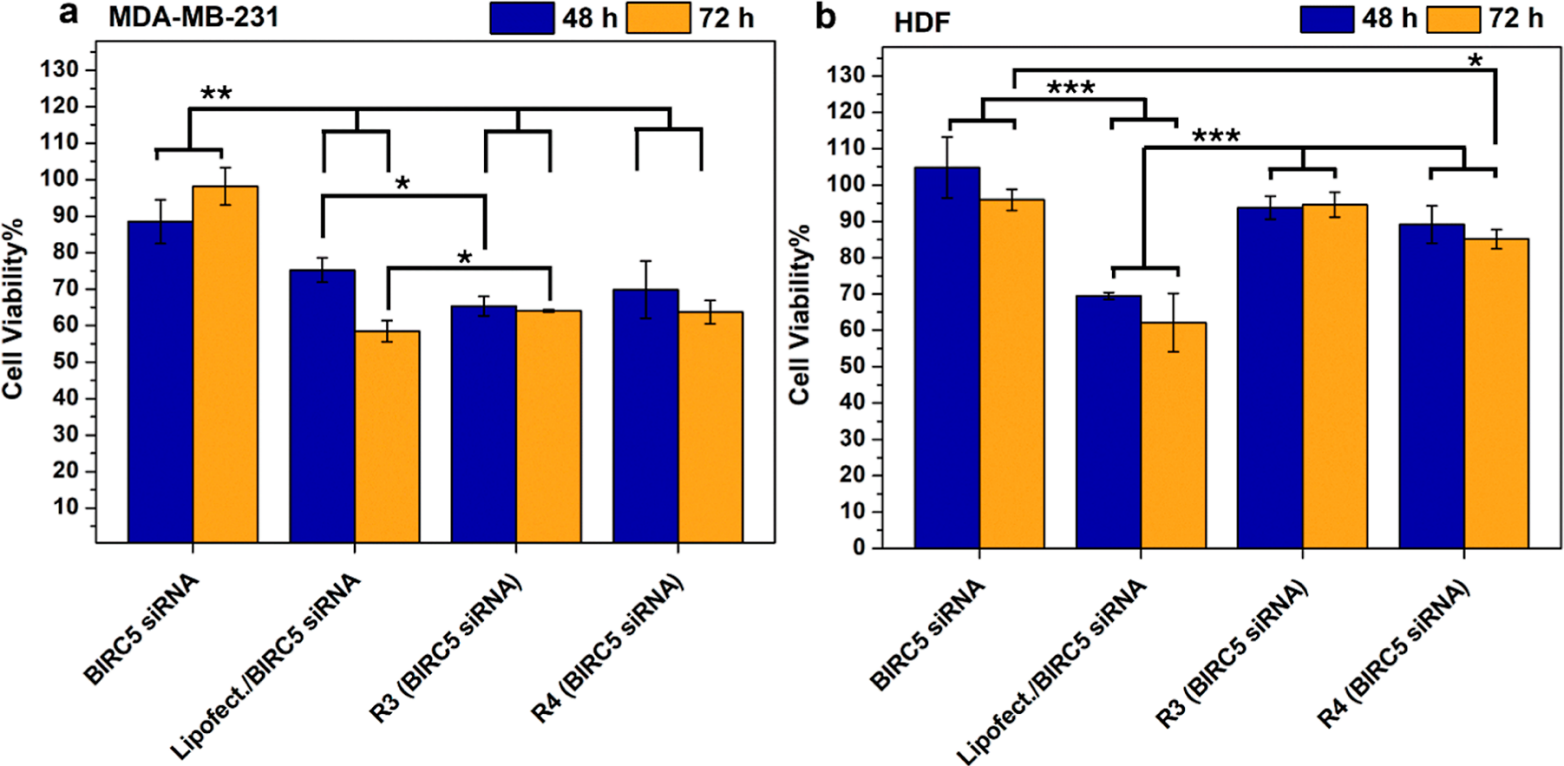
Cytotoxicity studies on MDA-MB-231 (a) and HDF (b) conducted on CDs-pDEAEMA/BIRC5 siRNA complexes at w/w ratios of 3 and 4, lipofectamine/BIRC5 siRNA, and naked BIRC5 siRNA, after 48 h (blue) or 72 h (orange) of incubation and expressed as percentage of viability with respect to untreated control, incubated with medium only.

To confirm that the cytotoxic effect was indeed due to the action of delivered siRNA, the same studies were conducted on HDF cells. As expected, in this case, all of the tested complexes were found to be cytocompatible. These findings are encouraging, highlighting that the therapeutic CDs-pDEAEMA/BIRC5 siRNA complexes effectively reduce the viability of MDA-MB-231 cancer cells while causing no cytotoxic effects on healthy HDF cells.

To verify the actual decrease in survivin production due to BIRC5 silencing, a specific ELISA assay was performed to quantify survivin levels in MDA-MB-231 cell cultures.

As shown in Figure 9, the data obtained are particularly encouraging. Specifically, both tested CDs-pDEAEMA-based complexes significantly reduced survivin production, silencing more than 80% of BIRC5. This finding is even more compelling considering that the difference is statistically significant compared to the action of both naked siRNA and the complex formulated with Lipofectamine. These results demonstrate that CDs-pDEAEMA/BIRC5 complexes can effectively achieve BIRC5 silencing in triple-negative breast cancer cell cultures.

**Figure 9:**
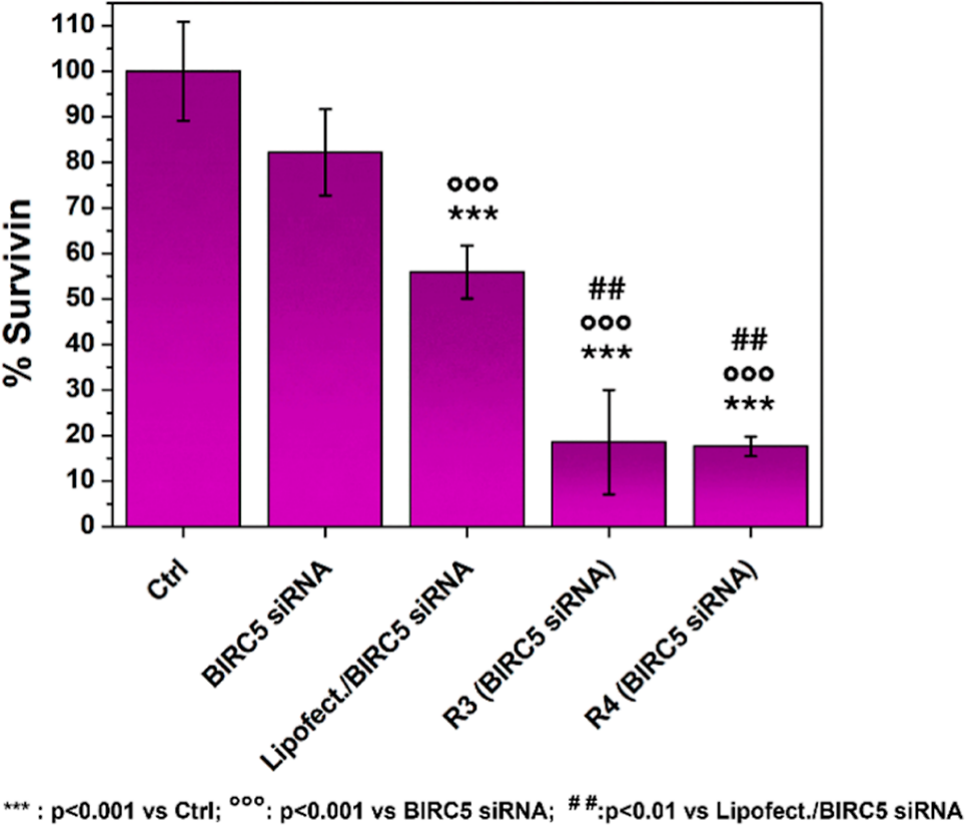
ELISA survivin assay on MDA-MB-231 lysates after 72 h incubation with CDs-pDEAEMA/BIRC5 siRNA complexes at w/w ratios of 3 and 4, lipofectamine/BIRC5 siRNA, and naked BIRC5 siRNA.

Finally, to assess the cellular internalization capabilities of the siRNA delivery system CDs-pDEAEMA, uptake studies were conducted on MDA-MB-231 and HDF after 4 h (Figures S2 and S3) and 24 h (Figures 10 and S4).

**Figure 10:**
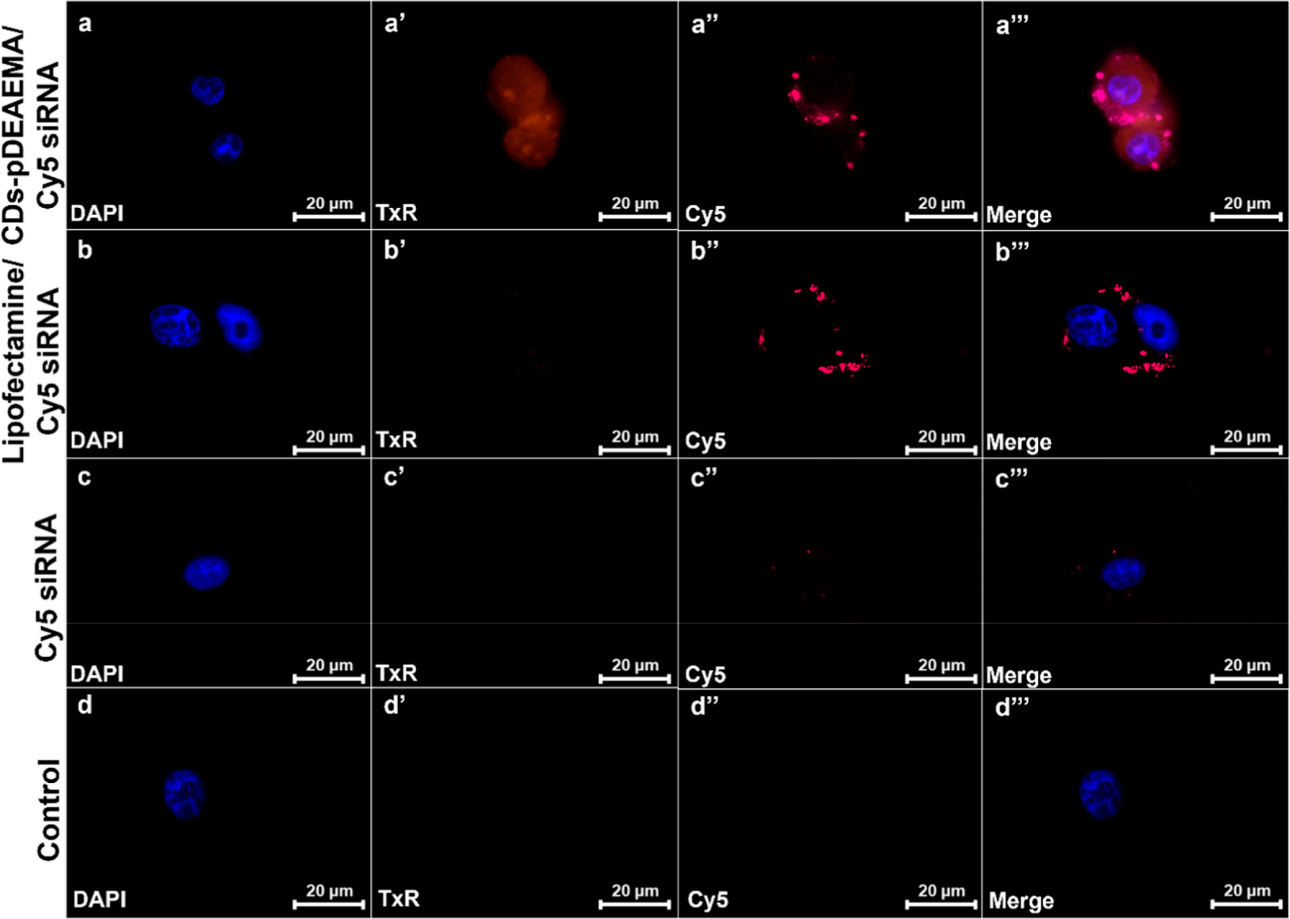
Uptake studies of CDs-pDEAEMA/Cy5-labeled siRNA. Fluorescence acquisitions in DAPI (blue), Texas Red (red), Cy5 channels (deep red), and merged micrographs following the uptake of CDs-pDEAEMA/Cy5 siRNA (a), Lipofectamine/Cy5 siRNA (b), naked Cy5 siRNA (c) after 24 h incubation with MDA-MB-231 cells, and comparison with untreated control (d).

For both cell lines, the uptake appeared to be time-dependent (Figures 10, S2, S3, and S4). After 24 h of incubation, a substantial internalization of siRNA delivered by the complexing agents CDs-pDEAEMA (Figure 10a′′′) and Lipofectamine (Figure 10b′′′) can be observed in MDA-MB-231 cells, as monitored through Cy5 fluorescence of the labeled nucleic acid. An even more intriguing result is shown in Figure 10a′′, captured in the Texas Red fluorescence channel. Specifically, widespread fluorescence throughout the cytoplasm is visible, attributable to the presence of CDs-pDEAEMA.

Thanks to the optical properties of CDs, which have been enhanced and optimized through passivation, enabling an expanded emission spectrum toward longer wavelengths, the complexes are easily detectable in the red region. This feature allows a potential in vivo diagnostic signal with reduced interference from tissues and biological components.^39,40^ Thus, the results of this study demonstrate that CDs-pDEAEMA complexes are efficiently internalized and can serve as fluorescence contrast agents, paving the way for potential real-time therapy monitoring through a theranostic approach.

The siRNA uptake was also investigated quantitatively, following the fluorescence signal of labeled siRNA after cell lysis (Figure 11.)

**Figure 11:**
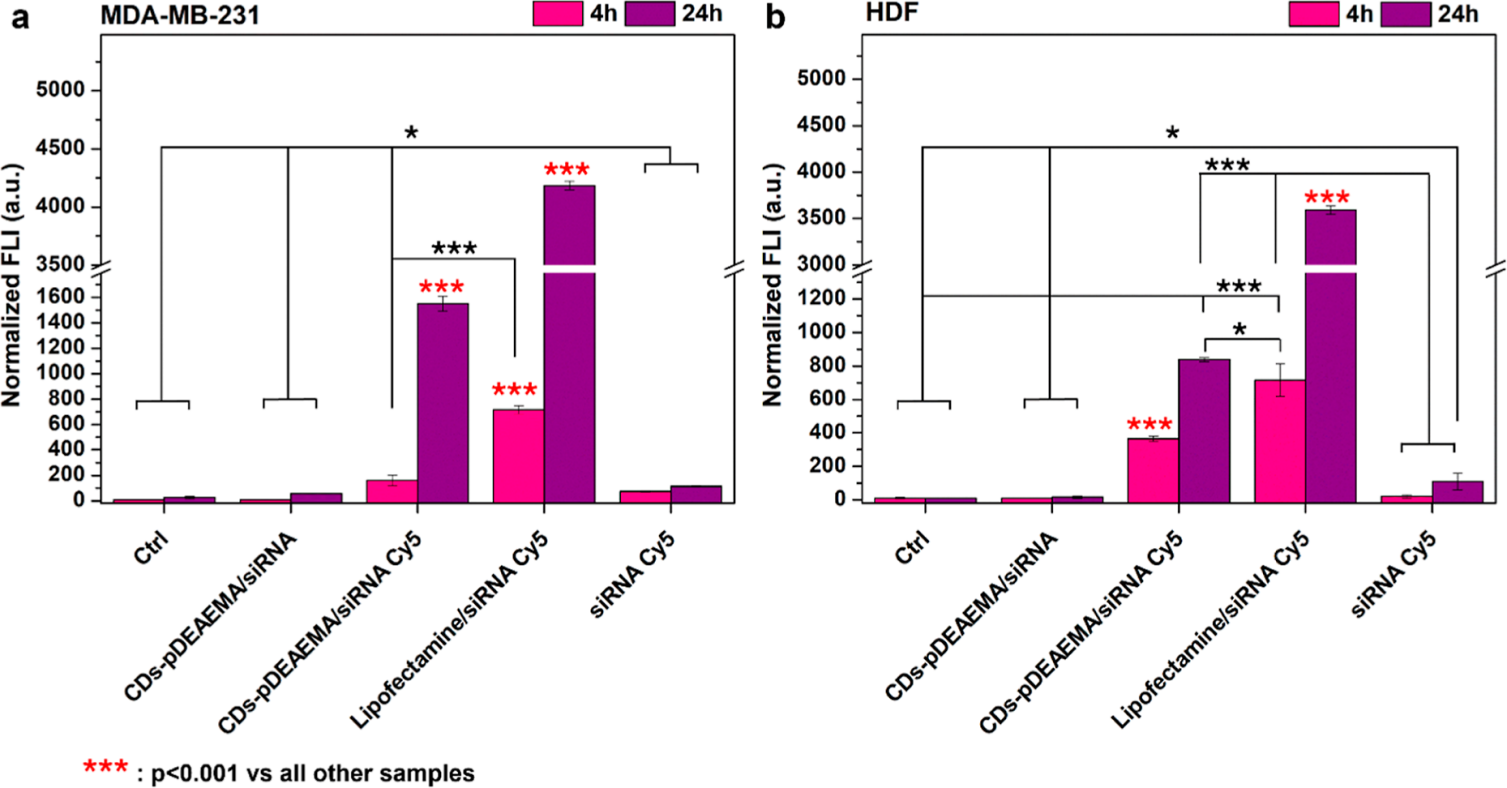
Quantitative analysis of Cy5-labeled siRNA uptake in MDA-MB-231 (a) and HDF (b) cells after 4 h (pink) and 24 h (purple) incubation with CDs-pDEAEMA/Cy5 siRNA, Lipofectamine/Cy5 siRNA, naked Cy5 siRNA compared to controls of untreated cells, and CDs-pDEAEMA/(non-fluorescent, scrambled) siRNA.

Even though the results align with the qualitative data acquired through microscopy, this study provided a deeper understanding with additional details. For instance, it appears that a small part of the free siRNA undergoes time-dependent internalization. Furthermore, as expected, the complexes with Lipofectamine, used as a positive transfection control, exhibit the highest internalization levels in both cell lines.

On the other hand, while CDs-pDEAEMA/Cy5 siRNA complexes do not reach the internalization levels of the positive control, they are still abundantly uptaken by both cell lines, although with some differences. Notably, in addition to a more pronounced time dependency in MDA-MB-231 cells, after 24 h, the CDs-pDEAEMA/Cy5 siRNA complexes appeared to be more internalized in cancer cells than in HDF. This finding is particularly encouraging as it suggests a potential selectivity for tumor cells after prolonged exposure.

Overall, the data confirm efficient internalization, coupled with the possibility of tracking therapy progression through fluorescence imaging in the red window, thanks to the presence of CDs.

## Conclusions

We designed a theranostic nanoplatform capable of delivering survivin-silencing siRNA potentially enabling targeted triple negative breast cancer (TNBC) treatment and monitoring. Biocompatible and highly fluorescent CDs were engineered through controlled polymerization (ATRP) of 2-(diethylamino)­ethyl methacrylate (DEAEMA) monomers to give CDs-pDEAEMA and providing improved optical and physicochemical properties to allow interactions with siRNA. The functionalization of these CDs resulted in an extension of CDs emission spectrum to the red region (∼620 nm), desired features for deep tissue penetration, and higher resolution imaging. The CDs-pDEAEMA nanocarriers exhibited excellent siRNA complexation capabilities and stability toward polyanionic exchange at low CDs-pDEAEMA/siRNA ratio (*R* = 3), suggesting no premature disassembling after administration. Furthermore, this hybrid complexing agent demonstrated to protect the siRNA cargo against degradation by RNAse A. To assess their biomedical performance, in vitro studies were conducted to evaluate their cytocompatibility, erythrocompatibility, and silencing efficacy of the nanocarriers. Erythrocompatibility tests confirmed their safety on red blood cells (5% and comparable to PBS), while viability assays demonstrated their compatibility with human dermal fibroblasts (HDF). Further in vitro characterization showed successful transfection of the TNBC cell line MDA-MB-231 with a decrease in cell viability and effective survivin silencing (80% reduction) after CDs-pDEAEMA/BIRC5 siRNA treatment. Moreover, the CDs-pDEAEMA/BIRC5 siRNA complexes can enter MDA-MB-231 cells and can be easily visualized by using fluorescence imaging in the red emission region. On the whole, these findings underscore the potential of CDs-pDEAEMA as advanced theranostic tools, capable of effective siRNA delivery and survivin silencing, while enabling real-time imaging by fluorescence imaging. Future research should focus on optimizing surface modifications to provide targeting specificity and optical imaging in the NIR I spectral window so as to confer selectivity and enhanced imaging capabilities for deep tissue imaging. Additionally, in vivo studies will be essential to confirm their therapeutic efficacy and biocompatibility in a physiological environment. By further refining these nanocarriers, CDs-pDEAEMA could become powerful allies for more effective and personalized treatments of aggressive BC subtypes such as TNBC.

## Supplementary Material



## Notes

^1^H NMR spectrum of CDs-pDEAEMA with ACN as internal standard, 4 h uptake studies on MDA-MB-231 cell line of CDs-pDEAEMA/siRNA Cy5 and controls, 4 h uptake studies on HDF cell line of CDs-pDEAEMA/siRNA Cy5 and controls, and 24 h uptake studies on HDF cell line of CDs-pDEAEMA/siRNA Cy5 and controls (PDF)
