# Supplementary material for: Poly(2-(diethylamino)ethyl methacrylate)-Functionalized Carbon Nanodots as Theranostic Platforms for siRNA Delivery and Survivin Silencing in Triple-Negative Breast Cancer
Source: Biomacromolecules. 2025 May 10;26(6):3666–79. doi: 10.1021/acs.biomac.5c00267 (PMC12152932; doi:10.1021/acs.biomac.5c00267)
Supplement: Supplementary file 1 [file bm5c00267_si_001.pdf]

Supporting Information

Poly(2-(diethylamino)ethyl methacrylate)-Functionalized Carbon Nanodots as Theranostic Platforms for siRNA Delivery and Survivin Silencing in Triple Negative Breast Cancer

Paola Varvarà[a], Gennara Cavallaro[a] and Nicolò Mauro[a]*

[a] Laboratory of Biocompatible Polymers, Department of "Scienze e Tecnologie Biologiche Chimiche e Farmaceutiche" STEBICEF, University of Palermo, Via Archirafi 32 90123 Palermo (Italy)

*Corresponding author: nicolo.mauro@unipa.it

Summary

a. ^{1}H NMR spectrum of CDs-pDEAEMA with ACN as internal standard (**Figure S1**)

b. 4 h uptake studies on MDA-MB-231 cell line of CDs-pDEAEMA/siRNA Cy5 and controls (**Figure S2)**

c. 4 h uptake studies on HDF cell line of CDs-pDEAEMA/siRNA Cy5 and controls (**Figure S3)**.

d. 24 h uptake studies on HDF cell line of CDs-pDEAEMA/siRNA Cy5 and controls (**Figure S4)**.

a. ¹H NMR spectrum of CDs-pDEAEMA with ACN as internal standard (Figure S1)

To quantify the w/w % of poly-DEAEMA in CDs-pDEAEMA, a known weight of CDs-pDEAEMA was weighed and dispersed in D_2O and subsequently added with a known volume of acetonitrile (ACN) (used as an internal standard this analysis enabled the quantification of the w/w percentage of poly-DEAEMA in the CDs-pDEAEMA sample using an internal standard (Figure S1), which was determined to be 78% w/w.). The quantification was then obtained using the following equation:

$$mmol\ int\ std \div \frac{\int H\ int\ std}{n°\ H\ int\ std} = mmol\ DEAEMA \div \frac{\int H\ DEAEMA}{n°\ H\ DEAEMA}$$

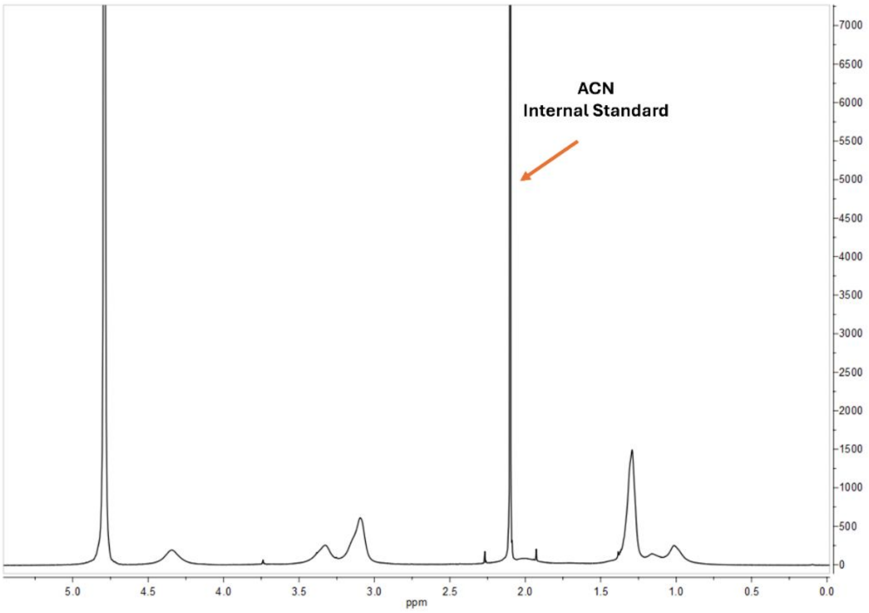

Figure S1. ¹H NMR spectroscopy of the derivative CDs-pDEAEMA using ACN as internal standard to quantify w/w% of poly-DEAEMA in CDs-pDEAEMA, D_2O, 400 MHz.

This analysis enabled the quantification of the w/w percentage of poly-DEAEMA in the CDs-pDEAEMA sample using an internal standard (Figure S1), which was determined to be 78% w/w.

b. 4 h uptake studies on MDA-MB-231 cell line of CDs-pDEAEMA/siRNA Cy5 and controls (Figure S2)

The qualitative cellular uptake of CDs-pDEAEMA complexes was investigated on MDA-MB-231 and HDF cell lines. Cells were seeded on 8-well plates (Glass Coverslips) at a density of 5 10^4 cells per chamber and maintained to adhere in complete DMEM at 37°C in a humidified environment for 24 h. Later, the medium

was discarded, the wells washed twice with sterile DPBS, and fresh OPTI-MEM containing CDs-pDEAEMA/siRNA Cy5 (R4), lipofectamine /siRNA Cy5, naked siRNA Cy5 or medium alone were incubated for 4 and 24 hours. Following the incubation period, the chamber contents were removed, the cells were fixed with a 4% formaldehyde solution in the same buffer (10 minutes, RT) and then the nuclei were stained with 4′,6-diamidino-2-phenylindole (DAPI) (10 minutes, RT). The wells were washed three times with DPBS both before and after fixation. Uptake micrographs were captured using a Zeiss fluorescence microscope, with images recorded by an Axio Cam MRm camera and a 100x magnification immersion objective using: DAPI (excitation: 359 nm, emission: 457 nm), Texas Red (excitation: 561 nm, emission: 594 nm) and Cy5 (excitation: 649 nm, emission: 670 nm) channels were used for all treated cell lines and experiments, ensuring consistent exposure times.

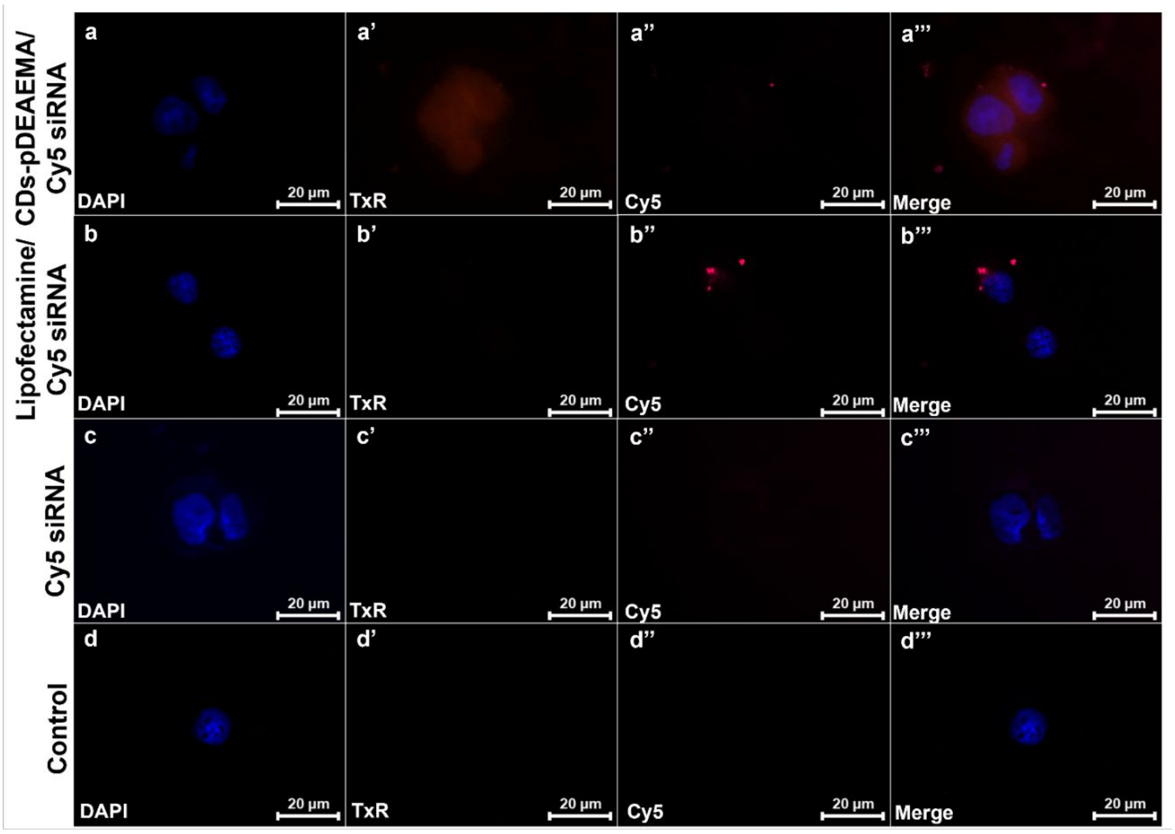

Figure S2. Uptake studies of CDs-pDEAEMA/Cy5 labelled siRNA. Fluorescence acquisitions in DAPI, Texas Red, Cy5 channels and merged micrographs following the uptake of CDs-pDEAEMA/Cy5 siRNA (a), Lipofectamine/Cy5 siRNA (b), naked Cy5 siRNA (c) after 4h incubation with MDA-MB-231 cells, and comparison with untreated control (d).

As shown in Figure S2, CDs-pDEAEMA/Cy5 siRNA begin to be internalized as early as 4 hours in MDA-MB-231 cells and can be observed both through red fluorescence (Figure S2 a’), which tracks the imaging

signal of the CDs, and in the siRNA-labeled channel, albeit to a lesser extent. In contrast, complexes formed with Lipofectamine, used as a transfection positive control, exhibit a significantly higher uptake (Figure S2 b'').

c. 4 h uptake studies on HDF cell line of CDs-pDEAEMA/siRNA Cy5 and controls (Figure S3).

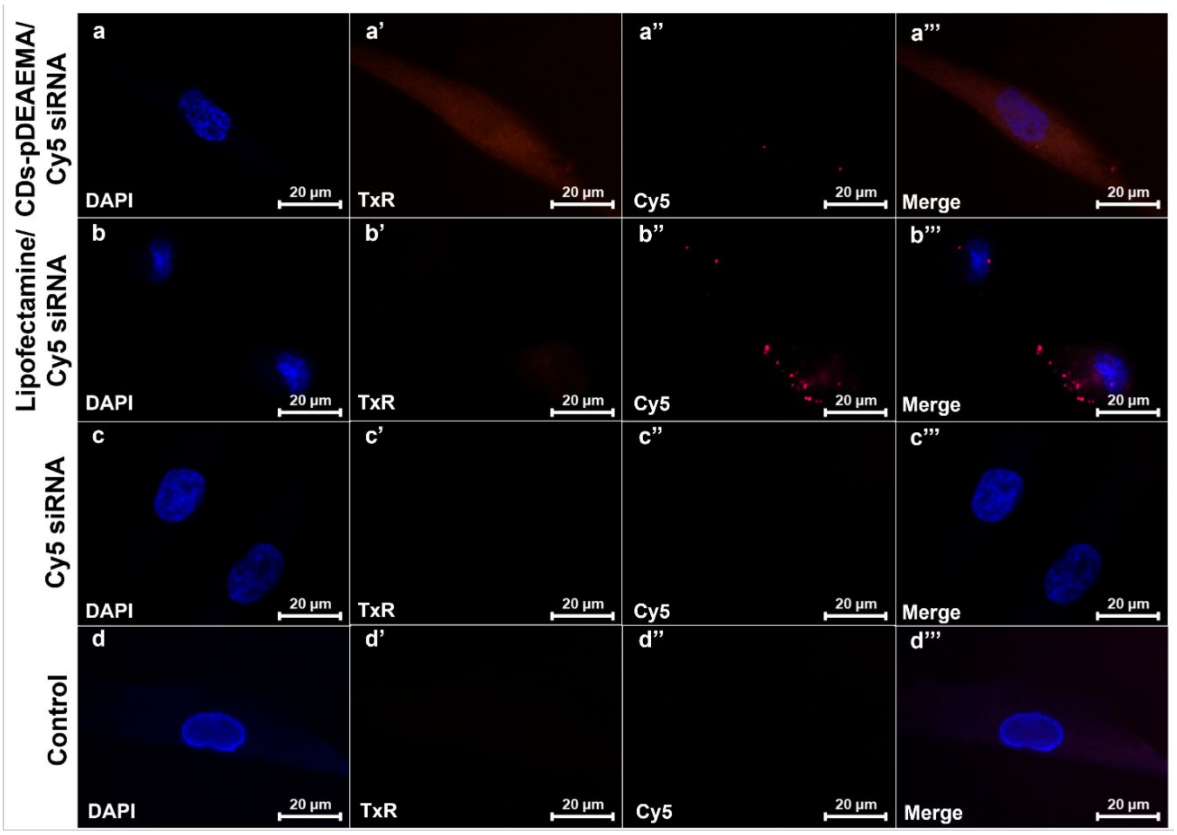

Figure S3. Uptake studies of CDs-pDEAEMA/Cy5 labelled siRNA. Fluorescence acquisitions in DAPI, Texas Red, Cy5 channels and merged micrographs following the uptake of CDs-pDEAEMA/Cy5 siRNA (a), Lipofectamine/Cy5 siRNA (b), naked Cy5 siRNA (c) after 4h incubation with HDF cells, and comparison with untreated control (d).

The uptake trend after 4 hours of incubation with the complexes was similar in HDF cells, with CDs-pDEAEMA/Cy5 siRNA easily visualized through red fluorescence (Figure S3 a') and a clear uptake of Lipofectamine/Cy5 siRNA (Figure S3 b''). However, a slight difference was observed after 24 hours.

d. 24 h uptake studies on HDF cell line of CDs-pDEAEMA/siRNA Cy5 and controls (Figure S4).

Although a time-dependent uptake was maintained in this cell line as well, CDs-pDEAEMA/Cy5 siRNA complexes (Figure S4 a') appeared to be less internalized in HDF cells than in MDA-MB-231 cells at the longer incubation time, showing a greater uptake difference compared to Lipofectamine/Cy5 siRNA (Figure S4 b''). This finding, confirmed by quantitative uptake studies (Figure 11), suggests a potential time-dependent selective effect.

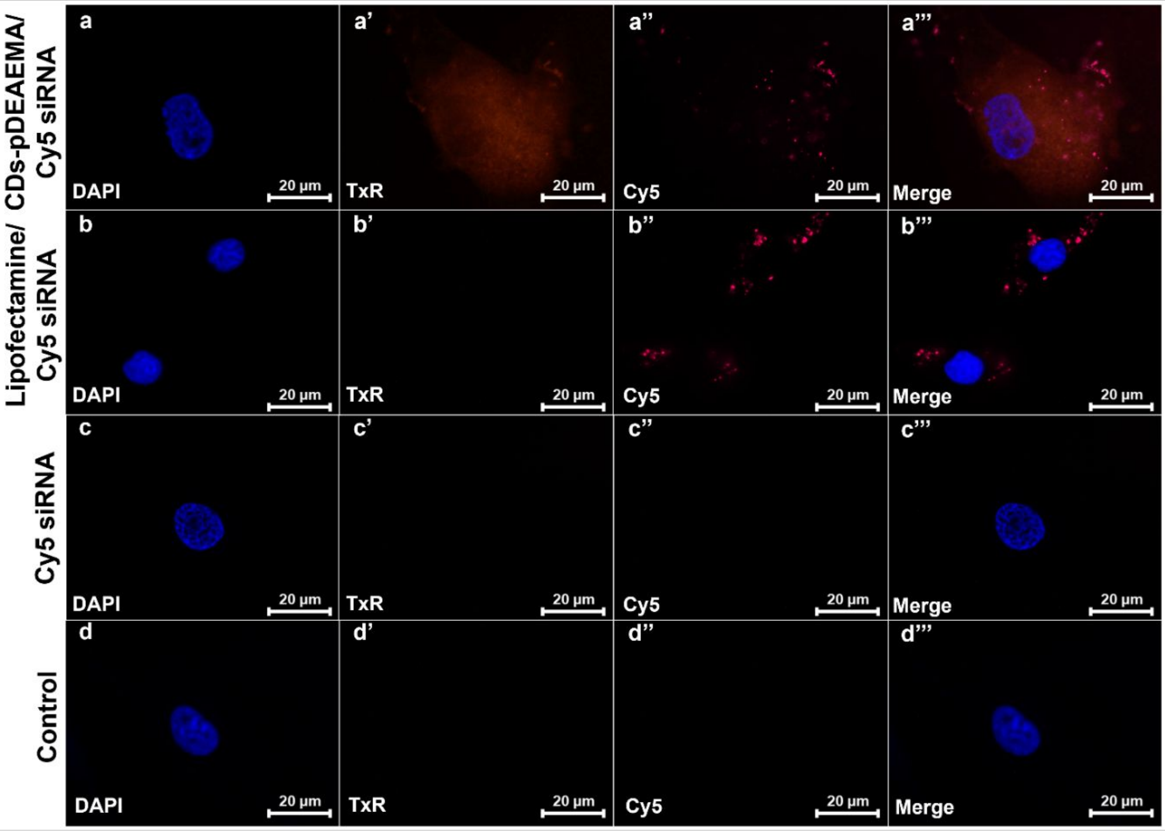

Figure S4. Uptake studies of CDs-pDEAEMA/Cy5 labelled siRNA. Fluorescence acquisitions in DAPI, Texas Red, Cy5 channels and merged micrographs following the uptake of CDs-pDEAEMA/Cy5 siRNA (a), Lipofectamine/Cy5 siRNA (b), naked Cy5 siRNA (c) after 24h incubation with HDF cells, and comparison with untreated control (d).
